# How to assess multimorbidity: a systematic review

**DOI:** 10.3389/fpubh.2025.1525593

**Published:** 2025-03-27

**Authors:** Li Yao, Qiaoxing Li, Yan Liu, Qinqin Li, Tingrui Wang, Zihan Zhou, Jiajia Yin

**Affiliations:** ^1^School of Management and Collaborative Innovation Laboratory of Digital Transformation and Governance, Guizhou University, Guiyang, Guizhou, China; ^2^Department of Respiratory and Critical Care Medicine, The Affiliated Hospital of Guizhou Medical University, Guiyang, Guizhou, China; ^3^School of Nursing, Guizhou Medical University, Guiyang, Guizhou, China

**Keywords:** multiple chronic diseases, multimorbidity, assessment methods, systematic review, old people, evaluation tools

## Abstract

**Objective:**

To comprehensively and systematically collect the methods used in the evaluation of patients with multiple chronic diseases both domestically and internationally, summarize and analyze the purpose, characteristics and validity of their initial development, and provide reference for health managers to choose appropriate evaluation methods for multiple chronic diseases.

**Methods:**

Analysis of the literature was based on searches conducted across eight electronic databases, including PubMed, EMBASE, Web of Science Core Collection, Scopus, Cochrane Library, CNKI, Wan Fang Database, and the Chinese Biomedical Literature Database (CBM). The initial search was completed on January 8, 2024, and the most recent update was conducted on December 10, 2024, with no restriction on the date of publication. The search process adhered to the 2020 PRISMA guidelines for systematic review.

**Results:**

54 literatures meeting the criteria were included, involving 54 evaluation methods of multiple chronic diseases. It can be divided into four categories: (1) assessment based on equal weight of disease count and disease severity; (2) based on physiological and psychological health status assessment; (3) evaluation based on drug use; (4) natural language processing evaluation system.

**Conclusion:**

Attention should be paid to the assessment of patients with multiple chronic diseases, and standardized and unified assessment methods should be developed in the future to expand the coverage of diseases and deepen the depth of assessment, so as to provide more comprehensive and accurate health management for the growing number of patients with multiple chronic diseases.

**Without patient or public contribution:**

This systematic review is primarily based on the comprehensive analysis of published literature and did not involve new data collection or direct participation of patients, hence there was no direct contribution from patients or the public.

**Systematic review registration:**

https://www.crd.york.ac.uk/prospero/, CRD42024530474.

## 1 Background

Multiple chronic diseases, as defined by the presence of 2 or more chronic conditions in a single individual, underscore the complexity and variety of health issues faced by individuals. These conditions can significantly impact an individual’s overall health, daily functioning, and quality of life (1). As populations age and the prevalence of chronic diseases rises, the challenge posed by multiple chronic diseases is increasingly becoming a global public health concern. Compared to a single chronic disease, having multiple chronic diseases not only results in more severe health consequences but also increases the complexity of disease treatment and health management. This situation is often linked to functional decline, disability, and premature death (2–4). From a public health perspective, this phenomenon drives total healthcare expenditures while exacerbating health inequities, as vulnerable populations experience earlier multimorbidity onset with poorer outcomes (5). Current clinical practice guidelines, however, remain predominantly single-disease focused, creating implementation barriers in real-world care settings (6).

Therefore, researchers are increasingly focusing on multiple chronic diseases, making related research a prominent topic in the field of health research. However, methodological issues in measuring multiple chronic diseases continue to be a challenge (7). Currently, there exists a range of tools for assessing multiple chronic diseases, however, there is a lack of standardized classification criteria. These tools are typically categorized into four groups: disease counting, organ- or system-based methods, weighted indices, and other assessment methods. While these tools are mainly utilized to gage the prevalence or pattern of multimorbidity, they can also be employed to forecast outcomes or assess interventions (8, 9). Researchers, healthcare providers, and policymakers encounter challenges when choosing the most suitable tool for assessing multiple chronic diseases. Factors such as effectiveness, reliability, feasibility, accessibility, cost, and ease of use of the assessment tool need to be considered.

In addition, the inconsistencies in defining terms related to multiple chronic diseases pose challenges in selecting appropriate assessment methods. The World Health Organization defines multiple chronic diseases as encompassing all conditions impacting an individual’s overall health, rather than focusing on a single disease (1). However, some assessment tools concentrate on comorbidities, which are other diseases co-existing with the primary condition. Different interpretations of ‘health status’ have resulted in various methods for measuring multiple chronic diseases, with debates on whether to incorporate acute illnesses, mental health issues, and so on (10–12). While, the standardization of the measurement of multiple chronic diseases is the basis for quantifying their symptom status, disease burden, treatment effect, etc. Comprehensive measurement studies of multiple chronic diseases are essential to understand their use requirements, advantages, limitations, and applications. This information can help health professionals and researchers to select appropriate measurement tools or develop new methods appropriate for specific health outcomes, thus enhancing the appropriate use of multiple chronic disease assessment tools. This systematic review aims to comprehensively examine the methods used for evaluating patients with multiple chronic diseases, both domestically and internationally. It summarizes and analyzes the objectives, characteristics, and validity of these methods’ initial development, critically assesses and compares their measurement attributes and quality of evidence, and discusses their interpretability and feasibility. The goal is to provide health managers with valuable insights to select appropriate evaluation methods for multiple chronic diseases, while also minimizing implementation barriers.

## 2 Methods

This review has been registered in the International Prospective Register of Systematic Reviews (PROSPERO registration number: CRD42024530474).

### 2.1 Eligibility

Inclusion criteria: (1) original research content focused on the development, testing, revision, and validation of multiple chronic disease assessment tools; (2) study types included cross-sectional, longitudinal, cohort, case–control studies and randomized controlled trials (RCTs).

Exclusion criteria: (1) comparative studies of multiple chronic disease assessment tools; (2) duplicate data collection; (3) unpublished or non-peer-reviewed literature, such as conference abstracts, preprints, policy papers, and informal publications; (4) inability to access the full text of the literature; (5) literature not in Chinese or English.

### 2.2 Search strategy

The methodology employed in this study is a systematic review that adheres to the PRISMA 2020 Guidelines for Systematic Reviews (13). We conducted comprehensive database searches across several platforms, including The Cochrane Library, PubMed, EMBASE, Web of Science Core Collection, Scopus, CNKI, Wan Fang Database, and the Chinese Biomedical Literature Database (CBM). These searches were performed without applying date restrictions. The initial search was completed on January 8, 2024, and the most recent update was conducted on December 10, 2024.

Before the official search, the research team first conducted a pre-search on PubMed and CNKI, then analyzed and discussed the search results, and adjusted the search strategy as needed to determine the official search terms. The retrieval terms used are ‘Multimorbidity,’ ‘Comorbidity,’ ‘Multiple Chronic Diseases,’ ‘Multiple Chronic Illnesses,’ ‘Multiple Chronic Medical Conditions,’ ‘Multiple Chronic Health Conditions,’ along with ‘Tool,’ ‘Instrument,’ and ‘Measure’. The search strategy incorporates a combination of subject words and free words, along with manual retrospective reference of included studies and relevant systematic reviews, reviews, and guides. All database search strategies are presented in Supplementary Boxes 1.

### 2.3 Selection of studies

The collected references were imported into EndNote 20 for literature management, where duplicates were eliminated. Two researchers, trained in systematic evidence-based methods, independently screened the literature in a structured manner based on predetermined inclusion and exclusion criteria. Initially, titles and abstracts were reviewed for screening, with literature failing to meet the criteria being excluded. Subsequently, full-text articles were reviewed.

In the process, the two reviewers also found 18 and 25 relevant articles from the references, respectively. The two reviewers then independently reviewed the 43 papers to ensure the rigor and objectivity of the selection process.

### 2.4 Data extraction

Data relevant to the research questions were extracted from the final set of included literature, such as developer, publication date, country/region, basic characteristics of the research subjects, tool name, and tool validity. Any discrepancies in the literature screening and data extraction process were resolved through discussion between the two researchers; if consensus could not be reached, a third researcher made the final decision.

### 2.5 Literature quality evaluation

Two researchers with evidence-based training conducted a qualitative evaluation of the literature across various types of studies, and cross-validated the evaluation results. Any discrepancies were resolved through consultation with a third researcher. The RCTs were assessed for risk of bias using the recommended tool from the Cochrane Handbook 5.1.0, with each study being evaluated independently. Studies that fully met the evaluation criteria were assigned Grade A, indicating the lowest probability of bias. Studies showing partial compliance were assigned Grade B, suggesting a moderate probability of bias. Studies with incomplete results, indicating the highest probability of bias and low study quality, were assigned Grade C. The quality of the cohort studies were assessed using the Newcastle-Ottawa Scale (NOS), which comprises three parts with a total of 8 items. These items include the selection of research subjects (4 items), between-group comparability (1 item), and exposure or outcome evaluation (3 items). The total score on this scale is 9 points, with studies scoring ≥7 being considered high-quality literature. Cross-sectional studies were assessed using the Australian JBI Evidence-Based Health Care Center (2016) quality evaluation tool, comprising a total of 8 evaluation items. Each item was assessed as “yes”, “no”, “unclear”, or “Not applicable”. The APPRAISE-AI Tool comprises 24 items with a cumulative score of 100. The tool categorizes quality as follows: very low quality (0–19), low quality (20–39), medium quality (40–59), high quality (60–79), and very high quality (80–100).

## 3 Results

### 3.1 Study selection

**Figure 1:**
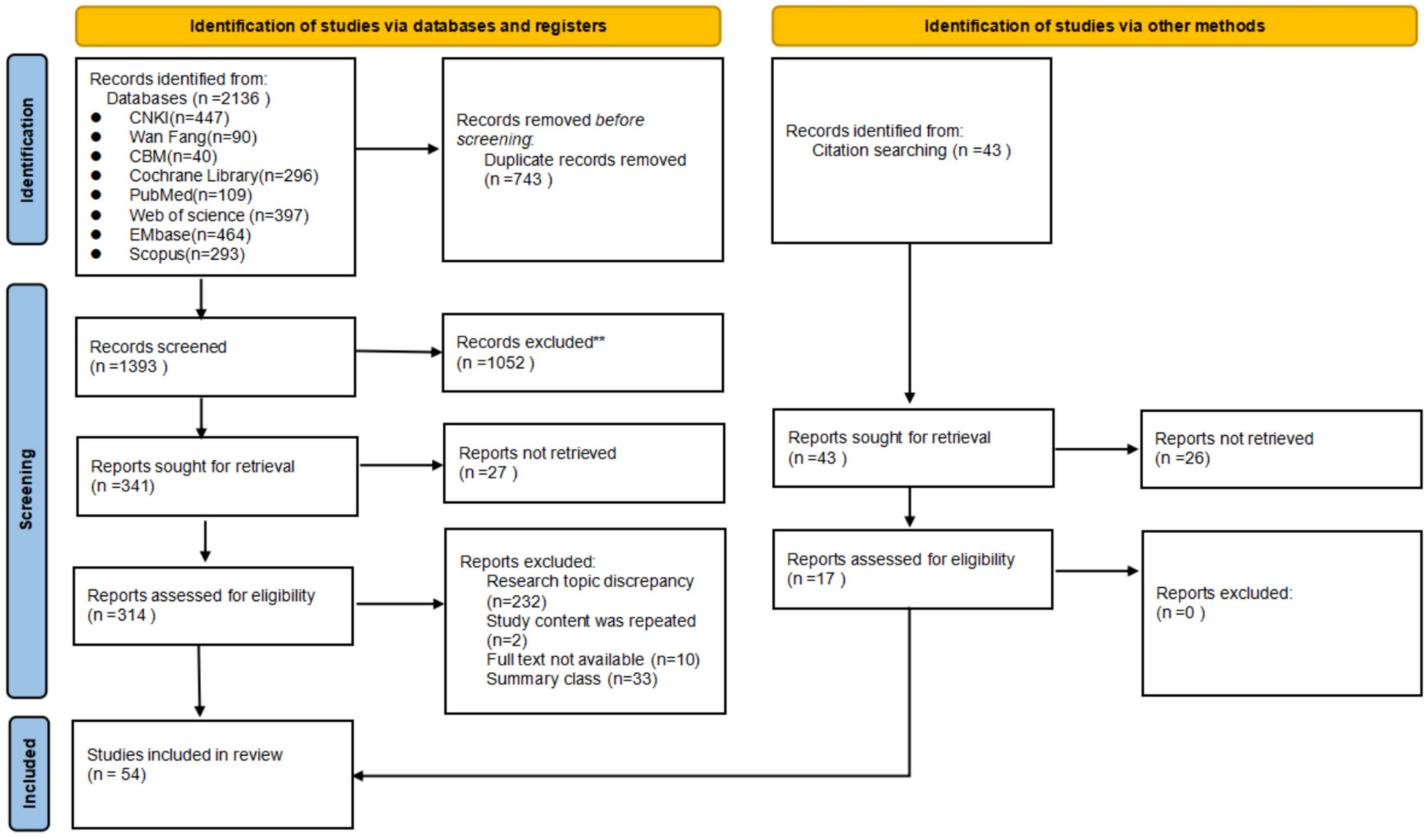
Flow diagram illustrating the original process of screening and identification of studies.

A total of 2,179 literature sources were identified during the initial search, with 1,436 remaining after the removal of duplicates. Following a thorough screening process based on predefined inclusion and exclusion criteria, two researchers independently reviewed the literature and reached a consensus on 54 relevant studies for final inclusion. The detailed process and outcome of literature screening can be seen in Figure 1.

### 3.2 Study characteristics

Fifty-four articles were published between 1968 and 2024, across 14 countries. The United States led with 27 articles, followed by Australia with five articles. Among the 54 studies, the development of multiple chronic disease assessment tools aimed to assess patient prognosis, disease status, and their impact and burden. The study participants in the included research encompassed a variety of groups such as community residents, inpatients, outpatients, and patients with specific diseases such as cancer and myeloma. Special groups such as patients with medical insurance, female community residents, and older patients were also included. Outcome indicators examined in the studies comprised mortality, hospitalization costs, medical resource utilization, and functional status. Refer to Table 1 for more details.

**Table 1:** Characteristics of included studies.

Study	Country /district	Year	Study purpose	Evaluation tools	Study object	sample	sex (Male/female)	Age	Data source	Outcome
Xu HW (36)	China	2024	Predicted disability trajectory	The Multimorbidity Index	Community resident	17,649	8,654/89,95	>50	Self-report	Disability change trajectory
Kar D (43)	UK	2024	develop and validate a modified version of CMMS	Modified Version of the Cambridge Multimorbidity Score	individuals with multimorbidity	500,000; 250,000	247,000/253,000; 123,500/126,500	>16	electronic medical records	mortality
Harrison H (44)	UK	2024	Implement and externally validate the Cambridge Multimorbidity Score	Cambridge Multimorbidity Score	adults aged	111,898	51,984/59,914	40–69	UK Biobank	Mortality, primary care consultation rate,cancer diagnosis
Shouval R (21)	USA	2022	Prediction of nonrecurrent mortality after allogeneic hematopoietic cell transplantation	The Simplified Comorbidity Index(SCI)	Hematopoietic cell transplantation patients	573	329/244	56(46–64)	Self-report, electronic medical records	Non-recurrent mortality,overall survival
Luo Y (45)	China	2022	develop a multimorbidity index incorporating disease combinations to predict 5-year mortality	Multimorbidity indices with individual diseases(MI), Multimorbidity Index incorporating Disease Combinations (MIDC)	older adults	11,853	6,287/5,566	≥65	Chinese Longitudinal Healthy Longevity Survey	5-year mortality risk
Hu WH (46)	China	2022	develop and validate a multimorbidity index for Chinese middle-aged and older communitydwelling individuals	the modified Chinese multimorbidity-weighted index (CMWI)	middle-aged and older people	20,035; 19,297	9,763/10,272; 8,769/10,528	>45	CHARLS, CLHLS	PF,ADL,IADL, mortality
Rotbain EC (22)	Denmark	2022	Assessing patient survival	The Chronic Lymphocytic Leukemia Comorbidity Index(CLL-CI)	Patients with chronic lymphocytic leukemia	4,975	3,030/1,945	70.7 (63.3, 78.1)	electronic medical records	Survival rate
Gensen C (23)	Netherlands	2022	Evaluate comorbidities in obese patients	The Metabolic Health Index(MHI)	Obese patient	11,501	10,003/498	45(21–64)	electronic medical records, National weight loss quality Registry data	Concentration of biorelated indicators
McEntee ML (39)	USA	2022	Improved methods for measuring polymorbiditis	Quality of Life Disease Impact Scale(QDIS)	Community resident	5,418	2,312/3,106	59.5 ± 13.7	Self-reported	Quality of life
Whitney DG (47)	USA	2021	Predicted mortality	Whitney Comorbidity Index(WCI)	Cerebral palsy	3,092	1,581/1,511	48 (18–89)	Insurance database	All-cause mortality
Wei MY (48)	USA	2021	developed and validated a multimorbidity-weighted index	multimorbidity-weighted index (MWI), Multimorbidity-weighted index ICD-coded conditions (MICD)	adults aged	18,212	7,923/10,289	>51	HRS, Medicare claims data	Mortality, future 8-year physical functioning
Berman AN (18)	USA	2021	Patients were evaluated for the presence of cardiovascular comorbidities	(The Cardio-Canary Comorbidity Project)	Cardiovascu-lar patient	1,000	—	—	electronic medical records	—
Spatola L (24)	Italy	2019	To evaluate the prognosis of patients with arteriovenous fistula	Subjective Global Assessment–Dialysis Malnutrition Score(SGA-DMS)	Hemodialysi-s patient	57	42/15	68.5 ± 14.5	Medical staff assessment	Thrombosis of vascular pathway, stenosis of vascular pathway
Wei MY (49)	USA	2018	How do chronic diseases or conditions affect physiological function	Multimorbidity Weighted Index(MWI)	Community resident	20,509	8,793/11,716	64.7 ± 10.7	Self-report	Activities of daily living function
Stanley J (50)	New Zealand	2017	Develop and validate short-term mortality risk indices	M3 Index	inpatient	3,331,811	1,592,493/1,739,318	>18	Health systems management database	mortality rate
Engelhardt M (14)	Germany	2017	To evaluate the prognosis of patients with myeloma	The Revised Myeloma Comorbidity Index(R-MCI)	Patients with myeloma	801	450/351	63 (21–93)	Medical staff assessment	lifetime
Fortin M (51)	Canada	2017	Assess the burden of multidisease	Self-Reported Chronic Disease Assessment Questionnaire	inpatient	367,713	154,311/213,402	52.3 ± 18.3	Self-report, electronic medical records	Prevalence rate
Corrao G (52)	Italy	2017	Develop and validate a novel comorbidity score	Multisource Comorbidity Score(MCS)	Community resident	500,000	—	≥50	Health systems management database	Mortality, Hospitalization, medical expenses
Fenollar-Cortés J (25)	Spain	2016	Assessing comorbidities in children with attention deficit/hyperactivity disorder	The ADHD Concomitant Difficulties Scale(ADHD-CDS)	Attention deficit/hyperactivity disorder children	696	429/267	11.65 ± 3.1	Parent report	Functional state
Thompson NR (53)	USA	2015	Assessing in-hospital mortality	Elixhauser-based Comorbidity Summary measure	inpatient	228,565	125,492/103,073	59.9 ± 18.7	electronic medical records	In-hospital mortality
Tonelli M (19)	Canada	2015	Use administrative data to identify the presence of chronic and multiple diseases	Tonelli Administrative Algorithms	Inpatient/out-patient	574,409	—	—	Health systems management database	Disease recognition efficiency
Dong YH (20)	Taiwan of China	2013	Assessing the risk of unplanned readmissions	The Pharmacy-Based Disease Indicator(PBDI)	inpatient	1,411,895	683,643/ 728,252	43.4 ± 16.4	Insurance database	Readmission rate
van Walraven C (33)	Canada	2009	Predicted in-hospital mortality	EI adaptation van Walraven	inpatient	345,795	172,897/172,898	58.0 ± 19.0	Insurance database	In-hospital mortality
Tooth L (54)	Australia	2008	To predict mortality rates and use of health services among older women	Weighted Multimorbidity Indexes	Community woman	10,434	0/10,434	73–78	Self-report	Mortality rate, use of health services, function of activities of daily living and quality of life
Newman AB (28)	USA	2008	To assess chronic disease status in older adults and predict mortality and disability	A Physiologic Index of Comorbidity	Medical insurance group	2,928	1,172/1,756	74.5	Self-report, electronic medical records	Mortality, level of mobility limitation, function of activities of daily living
Klabunde CN (26)	USA	2007	Predicting future treatment needs in the cancer population	CCI adaptation Klabunde	Cancer patient	140,315	85,967/54,348	>18	Insurance database	mortality rate
George J (55)	Australia	2006	Develop and validate a drug-based burden of disease index	Medication-Based Disease Burden Index(MDBI)	inpatient	317	—	71.8 ± 11.6	electronic medical records	mortality rate, Readmission rate
Groll DL (56)	Canada	2005	Develop an index of comorbidities as a result of physical function	Functional Comorbidity Index(FCI)	Spinal patient	37,772	17,854/19,918	18–103	Self-report	Quality of life, mortality rate
Byles JE (57)	Australia	2005	Predict mortality, hospitalization, etc.	The DVA PCT Multimorbidity Questionnaire	Veterans, war widows	1,303	482/821	≥70	Self-report	Disease severity, mortality rate, hospitalization rate
Bayliss EA (29)	USA	2005	Develop comorbidity assessment tools to quantify disease severity	Subjective Assessments of Comorbidity	Member of HMO Health Maintenance Organization	156	77/79	75	Self-report	Health status, physical function, Depression, self-efficacy
Sundararajan V (32)	Australia	2004	Predicted in-hospital mortality	New ICD-10 version of Charlson Comorbidity Index	inpatient	1,646,526	—	53.0 ± 21.0	Insurance database	mortality rate
Pope GC (58)	USA	2004	Projected medical costs	The CMS Hierarchical Condition Categories (HCC) Model(CMS-HCC)	inpatient	1,337,887	—	>18	Insurance database	Medical expenses
Sangha O (59)	Germany	2003	Developing a self-report-based comorbidity questionnaire and assessing its psychometric properties	The Self-Administered Comorbidity Questionnaire(SCQ)	inpatient	170	76/94	65.3 ± 8.8	Self-report	Hospitalization rate, acute hospitalization costs, quality of life
Fishman PA (60)	USA	2003	Identify chronic diseases and predict future health care costs	CDS adaptation RxRisk	Community resident	14,300,622	676,534/753,528	32.7 ± 19.6	Health service usage and cost database	Medical expenses
Rozzini R (61)	Italy	2002	To verify and compare the correlation between different comorbidities and disability in older patients	Geriatric Index of Comorbidity	Older patients	493	144/349	78.9 ± 7.4	Self-report, electronic medical records	mortality rate, Functional disability
Fan VS (62)	USA	2002	Assess comorbidities in the outpatient setting	Seattle Index of Comorbidity(SIC)	inpatient	10,947	10,659/288	≥50	Self-report	mortality rate, hospitalization rate
Miskulin DC (27)	USA	2001	To evaluate the comorbidity of hemodialysis patients	the Index of Coexistent Disease(ICED)	Hemodialysis patient	1,000	464/536	58 ± 14	Electronic medical record	mortality rate, hospitalization rate
Crabtree HL (63)	Britain	2000	To quantify the presence and severity of comorbidities in the older adults	The Comorbidity Symptom Scale (CmSS)	Older patients	183	58/125	>65	Self-report	Health status, anxiety, depression
Elixhauser A (34)	USA	1998	To predict hospital resource consumption and patient mortality	Elixhauser Index	inpatient	1,779,167	853,703/925,464	57.1	Insurance database	Hospital resource consumption, mortality rate
Incalzi RA (64)	Australia	1997	To evaluate comorbidities in older patients with acute medical diseases	Incalzi Index	Older patients	500	225/275	78.7 ± 5.9	Electronic medical record, Self-report	In-hospital mortality rate
Liu M (65)	Japan	1997	Assessing the functional status of stroke patients	Standardized Comorbidity Measures	Stroke patient	106	71/35	56.5 ± 13.2	electronic medical records	Functional impairment records
Shwartz M (66)	USA	1996	Assess medical expenses	Shwartz Comorbidity Scores	inpatient	4,439	—	≥65	electronic medical records	Patient cost
McGee D (67)	USA	1996	To assess the impact of multiple comorbidities on mortality	McGee Comorbidity Scores	Heart patient	13,247	5,383/7,864	49.64 ± 15.48	Self-report	incidence rate, mortality rate
Clark DO (15)	India	1995	Predict the frequency of use of medical resources, associated costs, and patient risk of death	Chronic Disease Score -Clark(CDS-Clark)	inpatient	250,000	185,250/64,750	≥18	Database of drug dispensing records	Medical expenses, frequency of visits
Greenfield S (68)	USA	1993	To verify the effect of comorbidity on the quality of life of patients	Four Level Index of Co-existent Disease(ICED)	Patients undergoing total hip replacement	356	43/313	64.0 ± 12.9	electronic medical records	Activities of daily living function
Romano PS (16)	USA	1993	Predicted risk of death	CCI adaptation Roman	inpatient	559	—	≥18	Insurance database	mortality rate
Parkerson GR Jr. (40)	USA	1993	Evaluate the health status and prognosis of patients	The Duke Severity of Illness Checklist(DUSOI)	Primary care patient	414	—	18–65	Medical staff assessment	Health state
Von Korff M (69)	USA	1992	Predicted mortality and hospitalization rates	Chronic Disease Score(CDS)	Community resident	122,911	—	≥18	Automated pharmacy database	mortality rate, Admission rate
Miller MD (70)	USA	1992	Assessing physical impairments in the older adults	Cumulative Illness Rating Scale-geriatric version(CIRS-G)	Older patients	141	—	≥65	electronic medical records	mortality rate
Deyo RA (71)	USA	1992	Predict patient prognosis	Deyo adaptation Charlson	inpatient	27,111	11,689/15,422	71.8	Insurance database	Postoperative mortality, complications, hospitalization costs
Weiner JP (72)	USA	1991	To predict the use of outpatient medical services	Ambulatory Care Groups(ACG)	out-patient	160,000	—	≥18	Insurance database	Number of visits
Charlson ME (37)	USA	1987	Comorbidity was assessed to predict mortality	Charlson Index	Breast cancer patient	559	—	≥18	electronic medical records	mortality rate
Kaplan MH (73)	USA	1974	To explore the health problems in the complications of diabetes	Kaplan-Feinstein Index	diabetic	201	201/0	54.5 (25–85)	electronic medical records	mortality rate
Linn BS (17)	USA	1968	Assess the patient’s physical impairment	Cumulative Illness Rating Scale(CIRS)	inpatient	20	—	≥18	electronic medical records	Bodily injury

—no reported; PF, Physical Functioning; ADL, Activities of Daily Living; IADL, Instrumental Activities of Daily Living.

### 3.3 Evaluate data source characteristics

Data sources for assessing multiple chronic diseases across the 54 articles included the following categories: (1) Electronic medical records; (2) Insurance database; (3) Self-report; (4) Public health databases; (5) Caregiver report; (6) Administrative database. The majority of studies, 13 in total, utilized data from electronic medical records. Six studies combined data from electronic medical records, self-reports, and other registration databases. Eleven studies relied solely on self-reports, while caregiver reports, including parent reports and medical staff assessments, were used in a total of four studies. Please refer to Table 1 for more specific information.

### 3.4 Basic characteristics of evaluation tools

**Figure 2:**
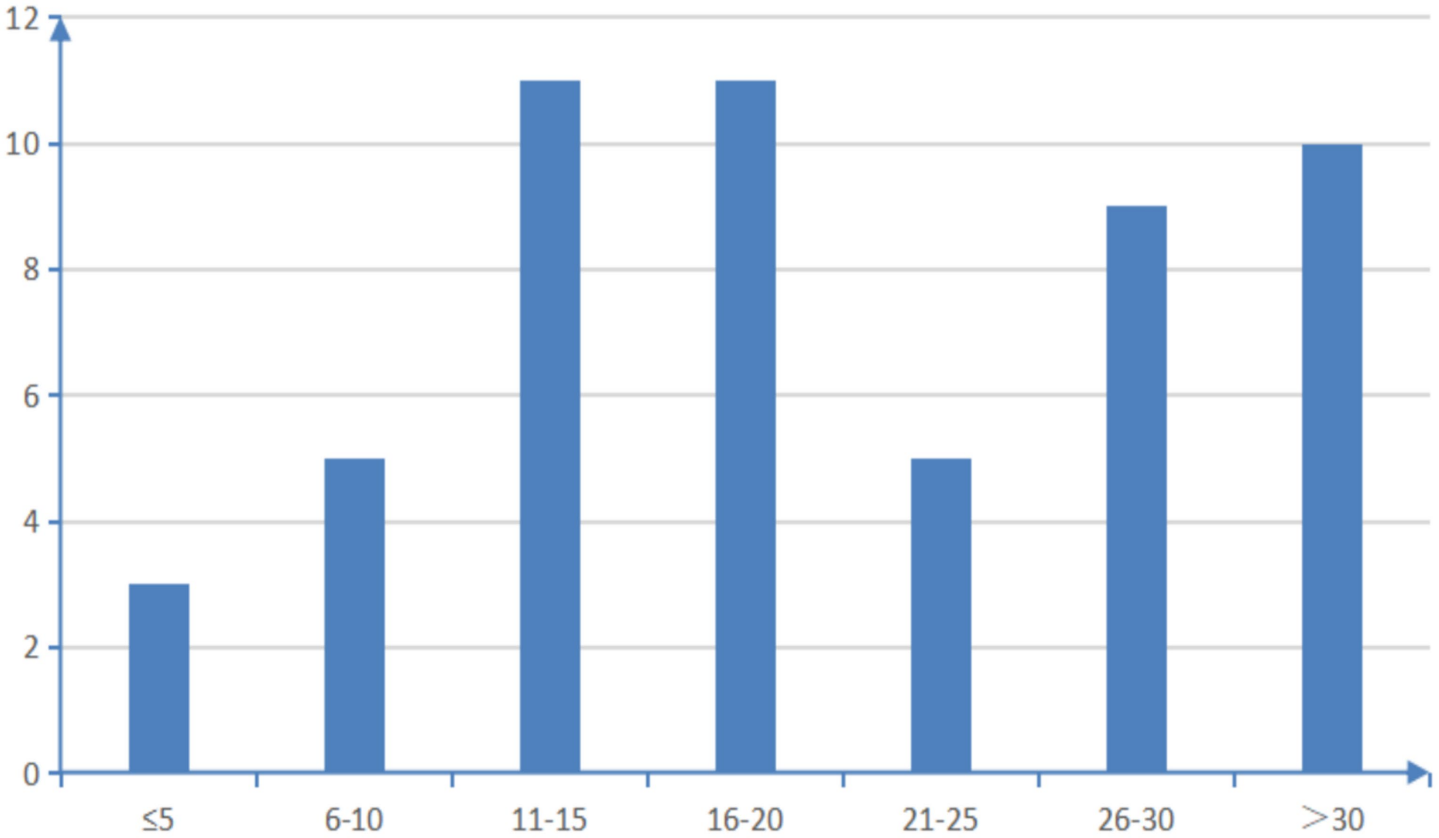
Overview of the number of types of diseases/health conditions covered by the assessment tools.

**Figure 3:**
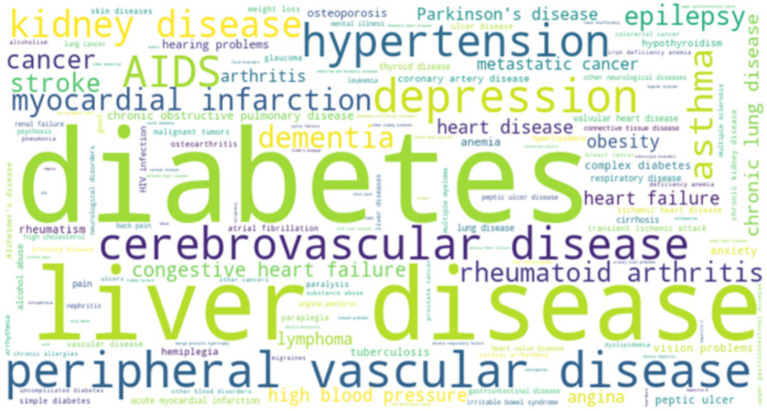
Cloud map of disease/health status words involved in the assessment tool.

A total of 54 multi-chronic diseases assessment tools were included in this study, categorized as follows: (1) assessment based on equal weight of disease count and severity; (2) assessment based on physiological and health status; (3) evaluation based on drug use; (4) natural language processing evaluation system. Among the 54 studies, we summarized the reliability and validity of all the evaluation tools as shown in Supplementary Table 1. Thirty-four studies examined the predictive accuracy of various chronic disease assessment tools on outcome indicators. Among them, eight studies were validated by comparing them with established multi-chronic diseases assessment tools like the Elixhauser index and the Charlson comorbidity index. Fourteen studies utilized methods such as the C-statistic to assess the effectiveness of the model. The 54 assessment tools evaluated in these studies covered a range of 4 to 70 disease/health status categories, with only one paper not specifying any particular disease/health status category. As depicted in Figure 2, 85.2% of the assessment tools addressed over 10 disease/health conditions. Supplementary Table 2 provides a detailed breakdown of the types of illnesses/health conditions included. To gain deeper insights into the disease/health status encompassed by the various multiple chronic disease assessment tools, the researchers utilized Python to generate a word cloud map (Figure 3).

### 3.5 Methodological quality of included studies

This study comprised 3 randomized controlled trials and 15 cross-sectional studies, all of which were rated above grade B in quality assessment. Among the 28 cohort studies, 21 scored ≥7 points on the NOS scale. The quality of the included literature was deemed high, as indicated in Supplementary Tables 3–5. The quality of the remaining 8 studies was evaluated using APPRAISE-AI, with 4 studies (14–17) of them being of medium quality. The quality of the included literature deemed medium, as indicated in Supplementary Table 6. Despite some bias related to subject grouping, follow-up duration, and control for confounding factors, such as the lack of a clear randomization method in the studies, all the research was deemed relevant for this review.

## 4 Discussion

Currently, research on multiple chronic diseases is extensively conducted worldwide, encompassing diverse populations and exhibiting significant diversity. Additionally, a broad spectrum of assessment tools for multiple chronic diseases exists, utilizing varied data sources. While most tools exhibit satisfactory reliability, validity, and adaptability in the evaluated populations, some tools still have certain limitations in their applicability. Consequently, existing research conclusions offer valuable reference and practical guidance for the assessment and management of multiple chronic diseases. However, further optimization of these tools is necessary to accommodate a broader range of application scenarios.

### 4.1 Multiple chronic disease assessment tools vary, but a common assessment method is lacking

As the population ages and lifestyles evolve, the prevalence of patients with multiple chronic diseases is on the rise. In order to optimize the management and treatment of these diseases, this study systematically reviewed multiple chronic disease assessment tools from the 1960s to the present, aiming to evaluate their applicability and effectiveness to patient health status and treatment outcomes in different clinical scenarios. These tools encompass weighted scoring systems, exponential classifications, and natural language processing models. With the popularization of electronic health records and the development of information technology, multiple chronic disease assessment tools are no longer limited to conventional assessment methods (such as disease-specific counting scales and functional status assessments). Some researchers began to integrate artificial intelligence and big data analysis technology to develop an intelligent multiple chronic diseases assessment system. However, the interoperability of these classification evaluation systems still needs to be further verified (10, 18–20). The variety of assessment tools for multiple chronic diseases reflects the intricate nature and varied manifestations of such conditions. These tools may vary in their focus on different risk factors, types of diseases, and prediction models to cater to diverse health management needs (1). For instance, certain tools excel in evaluating cardiovascular disease risk (18), while others are better suited for assessing metabolic syndrome, cancer, or other specific diseases (14, 21–27). This diversity allows for tailored and personalized assessments for particular populations, but it also leads to a wider range of assessment outcomes. To enhance the accuracy of assessment, healthcare personnel may utilize a combination of various assessment tools, including subjective assessment indexes and physiological indicators of comorbidity, to achieve more comprehensive and precise assessment outcomes (8, 28, 29). Although, the combination of different assessment tools can improve the accuracy and completeness of the assessment to some extent. But it also brings a series of challenges: first, it not only reduces the efficiency of assessment, but also requires medical staff to spend more time and effort to familiarize themselves with and understand the use of different assessment tools and the interpretation of results. Second, some assessment tools may require additional resources, such as specialized equipment and trained personnel, leading to a potential waste of medical resources and potential delays in diagnosis and treatment. Therefore, the feasibility and economy of the combined use of multiple chronic disease assessment tools need to be further considered (9). In addition, the absence of standardized evaluation methods hinders the advancement of personalized medicine. A generic assessment approach can effectively combine various types of information to precisely evaluate a patient’s risks and requirements, enabling the development of a more tailored treatment plan. Based on this, a universal assessment method needs to be developed to facilitate the comparison and synthesis of multiple chronic disease assessment tools. The evaluation of multiple chronic diseases should prioritize simplicity, standardization, data compatibility, patient friendliness, and interdisciplinary applicability. Tools should be developed based on reliable evidence and extensive data, taking into account the association and interaction between different diseases. It is important to establish a consistent scoring system, standardized risk calculation formulas, and comparable thresholds to ensure the consistency and comparability of assessment results (30).

### 4.2 The data sources of multiple assessment tools are diverse, and their reliability needs to be further verified

Consistent with previous systematic reviews, this systematic review reveals that data on multiple chronic diseases assessments are derived from diverse sources, including medical records, clinical assessments, patient or caregiver reports, public health databases, and administrative databases like insurance claims and health system records (8, 10, 12). Diverse data sources can help achieve personalized assessment, because different assessment purposes and study populations may tend to require different types of data. For example, if you want to accurately assess patient disease burden, an accurate insurance claims database will be the best choice. However, if you want to gain a deeper understanding of the patient’s disease, physical and psychological conditions and needs, based on the patient’s self-reported symptoms and lifestyle, you may obtain more information that is not recorded or coded in the relevant database (31). Furthermore, there are differences in the completeness and accuracy of different data sources. For example, medical records and clinical evaluations in electronic medical records provide detailed medical information but may be subject to subjective physician judgment and recording errors, and do not include undiagnosed health conditions in the patient or conditions not listed on the diagnostic list. Although public health surveys and patient self-reports may be influenced by recall bias, social expectation bias, and subjective assessment, they are portable and can be collected longitudinally throughout the patient’s life cycle, which helps reduce underreporting. In addition, this approach is highly aligned with the promotion of patient self-management, self-care, and a patient-centered healthcare model. Insurance claims data offer extensive data coverage and the flexibility to create various measurement tools, but they are also susceptible to coding and recording errors. In addition, some researchers argue that insurance claims data may be the most practical method currently available to comprehensively evaluate patients with multiple chronic diseases. This approach can provide the large sample sizes necessary to study populations with specific clinical conditions or rare outcomes (8). The insurance claims database contains codes for patient diagnoses, which can be connected to other databases like electronic health records and research data. For instance, the updated version of the Charlson Comorbidity Index utilizes insurance claims databases that rely on ICD and CPT codes to evaluate patients with multiple chronic diseases (32). In contrast, the Elixhauser Index was created using insurance claims databases as its primary data source (33, 34). Studies have highlighted a lack of consistency between the Charlson comorbidity index derived from patient self-reports and medical records. However, despite this inconsistency, the predictive capabilities of both sources remain similar (35). This underscores the importance of considering data sources when creating and utilizing multiple chronic disease assessment tools. A thorough assessment of the reliability of these data sources is essential for the effective and precise multidimensional evaluation of patients with multiple chronic conditions.

In addition, this study evaluated the reliability and adaptability of multiple multimorbidity indices and other related tools. Supplementary Table 1 summarizes the reliability, validity and applicability of these assessment tools. Overall, these tools demonstrated good reliability and adaptability in predicting health outcomes in older patients with multiple chronic diseases. However, some scales have certain limitations in adaptability. Future research needs to conduct empirical verification in larger samples to further evaluate the reliability and validity of these tools. It is also necessary to consider verification in different populations and regions to assess the wide applicability of these tools.

### 4.3 The set of multiple chronic disease assessment tools covers a wide range of diseases and health conditions, but its scope and depth need further enhancement

The multi-chronic diseases assessment tool aims to comprehensively evaluate multiple diseases and health states of patients, and provide scientific basis for extensive health assessment and treatment decisions by analyzing their relationships with related outcome indicators such as mortality, medical costs and quality of life. These tools typically include a range of chronic diseases, such as cardiovascular disease, diabetes, chronic obstructive pulmonary disease, etc., as well as physiological indicators, related health factors, and other health conditions, such as blood pressure, weight, lifestyle, and activity function. However, it is noted that many existing measures for managing multiple chronic conditions are more geared toward research rather than practical health management (36). The widely used Charlson comorbidity index or disease count is simple to assess and the data is easy to obtain, but it cannot comprehensively reflect the overall experience of multiple chronic diseases (37). Research suggests that factors beyond disease lists, such as social support, coping mechanisms, personal preferences, living environment, and economic status, should also be taken into account when evaluating patients with multiple chronic conditions (30, 38). These additional factors could play a significant role in the development and management of chronic diseases. Researchers have found that disease severity can be included in assessment tools and the subjective impact of the disease on the patient’s social, mental, and physical health can be incorporated into patient reports, such as the Duke Disease Severity Checklist and the Comorbidity Subjective Assessment Index (29, 39, 40). Some studies have also pointed out that simple disease counts are suitable for estimating the prevalence of multiple chronic diseases and examining their clusters or trajectories to explore multimorbidity patterns in more depth, while weighted measures are more suitable for related risk adjustment and outcome prediction of multiple chronic diseases. Therefore, ensuring the accuracy of disease markers is crucial to determine the number of diseases to include in an assessment tool (38). Looking at the current set of multiple chronic disease assessment tools, the diseases and health conditions they cover are diverse, but their breadth and depth require further research. Specifically, although the existing multiple chronic disease assessment tools include many common chronic diseases, the coverage of other chronic diseases may be lower, such as rare diseases or diseases of more specific groups, and the breadth of the assessment tools may be insufficient. Therefore, further research is needed to expand the breadth of assessment tools to include more comprehensive coverage of various chronic diseases and health states. Second, the depth of an assessment tool refers to the degree of detail in which each disease/health state is assessed. Existing multiple chronic disease assessment tools typically focus on basic factors like disease and health, encompassing basic physiological indicators and symptom evaluation (30). Nonetheless, it may be necessary to incorporate more detailed assessment indicators for each specific disease or health condition. For example, in cardiovascular disease assessment, in addition to basic blood pressure and heart rate measurements, consider including results from tests such as electrocardiograms and cardiac ultrasounds. Therefore, further optimization of multiple chronic disease assessment tools is essential through ongoing scientific research, interdisciplinary collaboration, and the application of technology. This will enable the provision of more comprehensive and accurate health management services to patients.

### 4.4 Future development of multiple chronic disease assessment tools

The prevalence of multiple chronic diseases is rising due to the aging population and changes in lifestyle. While these patients may have stable health status, disease progression and new health issues can impact their prognosis. Regular comprehensive assessments are essential to promptly detect any changes in the patient’s health status. For the management and treatment of patients with multiple chronic diseases, it is crucial to conduct thorough research on various assessment methods (38, 41). However, this study identified issues with the existing assessment tools for multiple chronic diseases, including the lack of standardized and universally accepted tools, as well as insufficient breadth and depth of assessment content. In order to build a universal, standardized, intelligent, interdisciplinary, concise and efficient multi-chronic disease assessment tool suitable for the disease spectrum of Chinese patients, it is first necessary to clarify the universal definition of multi-morbidity. Currently, the term “multiple chronic diseases” refers to an individual experiencing two or more chronic health conditions simultaneously (42). Nevertheless, there remains ambiguity regarding whether this definition should encompass various factors linked to multiple diseases, including psychosocial factors, physical risk factors, social networks, disease burden, medical resource utilization, and patient coping strategies (11). Therefore, continuing to carry out large-scale multi-morbidity model research will help understand the special status of patients with multiple chronic diseases and lay a theoretical foundation for constructing multiple chronic disease assessment tools with higher structural validity. The establishment of interdisciplinary teams in medicine, information technology, sociology, psychology, and data science, along with the utilization of advanced technologies like artificial intelligence, machine learning, and natural language processing to integrate diverse data sources for developing a comprehensive multi-chronic disease intelligent assessment tool with robust interoperability, is a key research focus for the future.

### 4.5 Summary of quality

This study analyzed a total of 15 cross-sectional studies and 28 cohort studies. The majority of these studies clearly defined the inclusion and exclusion criteria for their research subjects, which were in line with the criteria used in this study. When evaluated using the NOS standard, the cohort studies scored between 5 to 9 points, with 19 studies scoring 8 to 9 points. The 15 cross-sectional studies included in the analysis all received a grade higher than B, indicating a high overall quality of the studies included. The literature elaborates on the research methods and outcome indicators, which help mitigate recall bias and selection bias to some extent. Bias primarily stems from the grouping of research subjects, follow-up duration, and control of confounding variables. For instance, the study lacks a clear randomization method. In general, the quality of the studies was deemed satisfactory.

### 4.6 Strengths and limitations

In this study, various methods for assessing multiple chronic diseases were compared, providing valuable references for health managers in selecting appropriate evaluation techniques for multiple chronic conditions. In addition, this study searched 8 relevant databases with reliable and sufficient data sources. Limitations: primarily, only articles in English and Chinese were considered, excluding evidence published in other languages. Furthermore, the integrated tools come from different literature, and although the quality of the literature has been evaluated, the usability and reliability of the tools have yet to be demonstrated. Lastly, the outcome measures in the studies were reported in scale form, lacking objectivity.

## 5 Conclusion

This study critically examined various existing assessment tools for multiple chronic diseases and identified areas for improvement, including versatility, reliability of data sources, and coverage of disease/health conditions. To effectively meet the needs of the increasing number of patients with multiple chronic diseases, future research should focus on creating a universal, standardized, and interdisciplinary multi-chronic disease assessment method. Meanwhile, the reliability of data sources should be further assessed and the range and depth of assessment tools should be expanded. Interdisciplinary collaboration and the integration of advanced technology will be crucial in developing an effective and intelligent tool for assessing multiple chronic diseases. This tool will serve as a methodological and theoretical foundation for offering patients more comprehensive and precise health management services.

## Data Availability

The data that support the findings of this study are available from the corresponding author, upon reasonable request.
