# Supplementary material for: How to assess multimorbidity: a systematic review
Source: Front Public Health. 2025 Mar 27;13:1525593. doi: 10.3389/fpubh.2025.1525593 (PMC11983592; doi:10.3389/fpubh.2025.1525593)
Supplement: Supplementary file 1 [file Table_1.doc]

**Supplementary Boxes 1. Search Strategies for Eight Databases**

#1 "Geriatric Assessment"[Mesh] OR (Tool [Title/Abstract]) OR (Instrument [Title/Abstract]) OR (Measure [Title/Abstract])

#2 "Multimorbidity"[Mesh] OR (Multimorbidity [Title/Abstract]) OR (Comorbidity [Title/Abstract]) OR ("Multiple Chronic Diseases"[Title/Abstract]) OR ("Multiple Chronic Illnesses"[Title/Abstract]) OR ("Multiple Chronic Medical Conditions"[Title/Abstract]) OR ("Multiple Chronic Health Conditions"[Title/Abstract])

#3 #1 AND #2

**Supplementary Box 1A. PubMed Retrieval Strategy**

#1 TI= (Tool OR Instrument OR Measure）

#2 TI= (multimorbidity OR comorbidity OR "Multiple chronic diseases" OR "Multiple Chronic Illnesses" OR "Multiple Chronic Medical Conditions" OR "Multiple Chronic Health Conditions")

#3 #1 AND #2

**Supplementary Box 1B. Web of Science Retrieval Strategy**

#1 (TITLE (tool) OR TITLE (instrument) OR TITLE (measure))

#2 (TITLE (multimorbidity) OR TITLE (comorbidity) OR TITLE ("Multiple chronic diseases") OR TITLE ("Multiple Chronic Illnesses") OR TITLE ("Multiple Chronic Medical Conditions") OR TITLE ("Multiple Chronic Medical Conditions"))

#3 #1 AND #2

**Supplementary Box 1C. Scopus Retrieval Strategy**

#1 'assessment'/exp

#2 tool: ti,ab,kw OR instrument: ti,ab,kw OR measure: ti,ab,kw

#3 #1 OR #2

#4 'multiple chronic conditions'/exp

#5 multimorbidity: ti,ab,kw OR comorbidity: ti,ab,kw OR 'multiple chronic diseases': ti,ab,kw OR 'multiple chronic illnesses': ti,ab,kw OR 'multiple chronic medical conditions': ti,ab,kw OR 'multiple chronic health conditions': ti,ab,kw

#6 #4 OR #5

#7 #3 AND #6

**Supplementary Box 1D. Embase Retrieval Strategy**

#1 (Multimorbidity): ti,ab,kw OR (comorbidity): ti,ab,kw OR (multiple chronic diseases): ti,ab,kw OR (multiple chronic illnesses): ti,ab,kw OR (multiple chronic medical conditions): ti,ab,kw OR (multiple chronic health conditions): ti,ab,kw

#2 (tool): ti,ab,kw OR (instrument): ti,ab,kw OR (measure): ti,ab,kw OR (assessment): ti,ab,kw

#3 #1 AND #2

**Supplementary Box 1E. Cochrane Library Retrieval Strategy**

#1 SU= comorbidity + multiple chronic diseases + multiple chronic diseases + chronic comorbidities + combined chronic diseases

#2 SU= Assessment + measurement + scale + questionnaire + item + tool + assessment

#3 #1 AND #2

**Supplementary Box 1F. CNKI Retrieval Strategy**

#1 Title or Keywords: (Co-morbidity) OR Title or Keywords: (Multiple Chronic Diseases) OR Title or Keywords: (Multiple Chronic Conditions) OR Title or Keywords: (Chronic Comorbidities) OR Title or Keywords: (Co-occurring Chronic Diseases) OR Title or Keywords: (Chronic Disease Co-morbidity)

#2 Title or Keywords: (Assessment) OR Title or Keywords: (Measurement) OR Title or Keywords: (Scales) OR Title or Keywords: (Questionnaires) OR Title or Keywords: (Items) OR Title or Keywords: (Tools) OR Title or Keywords: (Evaluation))

#3 #1 AND #2

**Supplementary Box 1G. Wan fang Retrieval Strategy**

#1 "Co-morbidity" (common field: intelligence) OR "Multiple chronic diseases" (common field: intelligence) OR "Multiple chronic conditions" (common field: intelligence) OR "Chronic comorbidities" (common field: intelligence) OR "Coexisting chronic conditions" (common field: intelligence) OR "Chronic disease comorbidity" (common field: intelligence)

#2 "Assessment" [common field: intelligence] OR "Measurement" [common field: intelligence] OR "Scale" [common field: intelligence] OR "Questionnaire" [common field: intelligence] OR "Item" [common field: intelligence] OR "Tool" [common field: intelligence] OR "Evaluation" [common field: intelligence]

#3 #1 AND #2

**Supplementary Box 1H. CBM Retrieval Strategy**
