# Supplementary material for: How to assess multimorbidity: a systematic review
Source: Front Public Health. 2025 Mar 27;13:1525593. doi: 10.3389/fpubh.2025.1525593 (PMC11983592; doi:10.3389/fpubh.2025.1525593)
Supplement: Supplementary file 2 [file Table_2.doc]

**Supplementary Table 1** Characteristics of evaluation tools

| **Number** | **Evaluation tools** | **Tool characteristics** | **Evaluation dimension** | **Comprehensive evaluation** |
| --- | --- | --- | --- | --- |
| 1 | The Multimorbidity Index | The disease index is divided into six grades and calculated by the sum of weighted scores | Predictive ability of multiple comorbidity trajectories | The Pearson correlation coefficient of multimorbidity index and disease count is 0.92, the C-statistic for the multimorbidity index was 0.739, and it has good applicability |
| 2 | Modified Version of the Cambridge Multimorbidity Score | Based on a high prevalence rate or high predictive value in the RSC data set | Chronic physiological function status | The Harrell C index was used to assess the differentiation of the models, with a Harrell C index of 0.92 for the risk of death, and it has good applicability |
| 3 | Cambridge Multimorbidity Score (CMS） | A multiple chronic disease scoring system based on primary care records to predict deaths, hospital admissions, and primary care consultation rates | Chronic physiological function status | The C-index values of 0.65 for death and 0.67 for primary care consultation rate suggest that the CMS has certain validity and reliability, the adaptability is limited |
| 4 | The Simpliﬁed Comorbidity Index（SCI） | The SCI index was composed of four comorbidities and age that predicted nonrecurrent mortality | Prediction of nonrecurrent mortality from multiple comorbidities in allogeneic hematopoietic cell transplantation | The AUC values were 70.3 and 72.0 in the development and external validation cohorts, respectively, which are higher than those of the traditional (HCT-CI). But there is no clear internal consistency coefficient for reliability, and the adaptability has certain limitations. |
| 5 | Multimorbidity Index（MI），Multimorbidity Index incorporating Disease Combinations (MIDC) | To predict 5-year mortality risk based on the number of chronic diseases reported by an individual; | To predict mortality in community-living elderly Chinese, and the effect of disease combinations on mortality | The Cronbach's alpha coefficient of MIDC is 0.65, and the C statistic of MIDC was 0.713, the adaptability is limited |
| 6 | Chinese Multimorbidity-Weighted Index（CMWI） | The multiple chronic disease burden is quantified by assigning weights to different chronic diseases | Chronic disease and chronic health function status | The prediction accuracy of ADL disability was 0.80, there is no clear internal consistency coefficient for reliability, the adaptability has certain limitations. |
| 7 | The Chronic Lymphocytic Leukemia Comorbidity Index（CLL-CI） | CLL-CI divides patients into low-, medium-, and high-risk groups, rapid assessment of comorbidities in newly diagnosed CLL patients and those requiring treatment, and is simple to implement. | Assessment of organ function | CLL-CI has good validity, there is no clear internal consistency coefficient for reliability, the adaptability has certain limitations. |
| 8 | The Metabolic Health Index（MHI） | MHI is based on objective parameters, on a scale from 1 to 6 | Human metabolic health | MHI has good validity, there is no clear internal consistency coefficient for reliability, the adaptability has certain limitations. |
| 9 | Quality of Life Disease Impact Scale（QDIS） | Single factor disease-specific measurement model allows each disease to receive a summary score | Chronic disease state | QDIS has satisfactory reliability, validity and adaptability |
| 10 | Whitney Comorbidity Index（WCI） | Added 7 new comorbidities that were not CCI or ECI, using Cox regression and competing risks analysis | Chronic disease state | WCI used Cox regression statistical analysis with good internal consistency, and the C-statistic values for predicting 1-year and 2-year mortality were 0.81 to 0.88, respectively, but the adaptability is limited |
| 11 | Multimorbidity-Weighted Index, （MWI），Multimorbidity-weighted index ICD-coded conditions (MICD) | The weight was assigned according to the impact of chronic disease on physical function, and the absolute value was combined to form an individual MICD score | Chronic disease and chronic health function status | The regression coefficient (β= -15.47) and determination coefficient (R²= 0.15) of MICD were better than Elixhauser and simple disease count, and the reliability and adaptability were better |
| 12 | The Cardio-Canary Comorbidity project | 5 different natural language processing (NLP) modules developed to assess cardiovascular comorbidities | Disease status | Cohen's Kappa value in the test set was 0.97, and the validity and adaptability of the scale were also good |

| **Number** | **Evaluation tools** | **Tool characteristics** | **Evaluation dimension** | **Validity evaluation** |
| --- | --- | --- | --- | --- |
| 13 | Subjective Global Assessment–Dialysis Malnutrition Score（SGA-DMS） | The SGA-DMS assesses 7 items, with scores ranging from 7 (normal) to 35 (severe malnutrition） | Nutrition and disease status | Age and CCI were positively correlated with SGA-DMS (B = 0.06, p = 0.02 and B = 0.31, p = 0.05). The area under the curve (AUC) of SGA-DMS is significantly higher than that of CCI and the modified CCI (0.70 vs 0.61 and 0.55) has good reliability and validity and feasibility |
| 14 | Multimorbidity Weighted  Index（MWI） | Based on the physiological function score of the SF-36 scale, a weighted comprehensive index was calculated for all diseases | A chronic health condition | The MWI scale performed well in reliability, validity and adaptability |
| 15 | M3 index | The M3 index has better predictive performance of mortality risk within one year than the Charlson and Elixhauser indices, and can better adjust the mortality risk caused by chronic diseases | Acute Chronic Pathophysiological Assessment | Compared with Charlson, the median difference in c-statistics = 0.009,（95%CI :0.008, 0.011）; the difference in M3 index compared with Elixhauser = 0.009 (95%CI: 0.007, 0.010), and it has good applicability |
| 16 | The revised Myeloma Comorbidity Index（R-MCI） | R-MCI is a 9-point scoring system that divides patients into 3 groups: good, moderate, and frail | Physiological function status | Comparing R-MCI with other risk scoring systems, it was found that its Brier score was the smallest, indicating smaller prognostic prediction errors and better reliability and validity, and it has good applicability |
| 17 | Self-Reported Chronic Disease Assessment Questionnaire | Total number of diseases and/or proportion of affected patients | Chronic physical and mental health conditions | The self-reported chronic disease scale performed well in terms of reliability, validity and adaptability |
| 18 | Multisource Comorbidity Score（MCS） | Phase weights are assigned according to the regression coefficients of the survival model to form an index consisting of 34 variables | Chronic diseases and health conditions | The higher the MCS value, the higher the incidence of these health outcomes, the better the scale reliability and validity, and it has good adaptability |
| 19 | The ADHD Concomitant Difﬁculties Scale（ADHD-CDS） | Brevity, help doctors design more comprehensive treatment targets | Health and functional status | Cronbach's alpha coefficient of this scale was 0.94, and ROC curve analysis showed AUC=0.979 (95% CI = [0.969, 0.989]). |
| 20 | Elixhauser-based Comorbidity Summary Measure | Weighted summary score based on the Elixhauser Comorbidity Index | A chronic health condition | The scale has good reliability and validity, with the model's c-statistic value being 0.804, and its adaptability is also good. |
| 21 | Tonelli Administrative Algorithms | Develop algorithms based on validation of ICD-9 CM/ICD-10 data to determine the presence or absence of 40 diseases. Those with positive predictive value and sensitivity ≥70% were rated as "high effectiveness"; those with positive predictive value ≥70% and sensitivity <70% were rated as "moderate effectiveness" | Chronic disease state | The scale can effectively distinguish the coexistence of multiple diseases, and has good internal consistency and adaptability |
| 22 | The Pharmacy-Based Disease Indicator（PBDI） | Developed based on chronic disease scoring framework and anatomic treatment chemical classification system, divided into five levels | Chronic physical conditions and drug use | The agreement between the two pharmacists on the PBDI total score was very high (ICC=0.99) and showed highly significant test-retest reliability (ICC=0.98) |
| 23 | EI adaptation van Walraven | Weighted additive index based on 30 chronic health states | A chronic health condition | The scale is built on the basis of multivariate logistic regression model, which ensures its good internal consistency, C statistic value is 0.760, and has good adaptability |
| 24 | Weighted Multimorbidity Index | Multimorbidity index based on self-reported data, predicting different outcomes by weighted and unweighted multimorbidity index | Physiological function status | The scale is constructed based on multivariate regression analysis, which ensures its good internal consistency, and has good validity and adaptability |
| 25 | A Physiologic Index of Comorbidity | Combined indicators of previous checks | Chronic disease state | The scale had good internal consistency and adaptability, and patients with an index of 7-10 had a hazard ratio of mortality of 3.80 (95% CI: 2.82-5.13) compared with those with a score of 0-2. |

**Continue to Supplementary Table 1**

**Continue to Supplementary Table 1**

| **Number** | **Evaluation tools** | **Tool characteristics** | **Evaluation dimension** | **Validity evaluation** |
| --- | --- | --- | --- | --- |
| 26 | CCI adaptation  Klabunde | A weighted index constructed by combining inpatient and outpatient claims data using weights from the original Charlson Comorbidity Index (CCI) | A chronic health condition | The scale is built on the basis of multiple independent health indicators, which can effectively distinguish different health outcomes and has good applicability |
| 27 | Medication-Based Disease  Burden Index（MDBI） | If a patient's medication regimen includes any drug listed in the MDBI, the patient will receive a disability weight for the appropriate disease condition | Disease status and drug use | There was significant correlation between MDBI score and CDS score (r=0.53; P<0.001), the ICC value is 0.98, and it has good adaptability |
| 28 | Functional Comorbidity Index（FCI） | The FCI is a simple count (yes/no) and a diagnostic weighted count, with the "weight" derived from the standardized beta coefficient of the regression analysis | Functional state | The FCI has intrinsic consistency and adaptability, and is better at explaining the variance of body function (R²=0.29) than the Charlson index (R²=0.18) and Kaplan-Feinstein index (R²=0.07). |
| 29 | The DVA PCT Multimorbidity Questionnaire | The DVA PCT multimorbidity questionnaire consists of 25 conditions | Health state | MI has good performance in content validity and criterion association validity, and has good adaptability, but the reliability evaluation of the scale needs further research |
| 30 | Subjective Assessments of Comorbidity | Calculate the sensitivity and specificity of each case relative to chart review, validating this newly developed tool against the assumed "gold standard" of chart review | Physical and mental health status | The scale has good reliability and validity. The median sensitivity and specificity relative to chart review were 75% and 92%, respectively. but its adaptability is limited |
| 31 | New ICD-10 version of The Charlson Comorbidity Index | Weighted index for 12 disease conditions based on original Charlson Comorbidity Index (CCI) weights and study-specific weights | Chronic physiological function status | The scale has good reliability and validity, and the C statistic is 0.855, but its adaptability is limited |
| 32 | The CMS Hierarchical Condition Categories model(CMS-HCC） | Regression analysis was performed using diagnostic data that included disease combination, age, and sex factors | A chronic health condition | The scale has good reliability and validity, and In predicting medical costs, the R^2^ value of the CMS-HCC model was 9.8%, but its adaptability is limited |
| 33 | The Self-Administered Comorbidity Questionnaire（SCQ） | SCQ items are short, easy to understand, and provide a sense of the severity of each comorbidity and its impact on functioning | Disease status and severity | The re-test reliability of SCQ was high, the Intraclass Correlation Coefficient was 0.94, and the Spearman correlation coefficient between SCQ and Charlson index was 0.32, showing good adaptability |
| 34 | CDS adaptation RxRisk | Chronic disease category algorithm based on prescription drug filling | Chronic physical and mental health functioning | The scale has good reliability and validity, and The RxRisk model explained 8.7% of the variation in health care costs, but its adaptability is limited |
| 35 | Geriatric Index of Comorbidity | The senile comorbidity index was divided into four levels | Disease severity and nutritional status | The Kappa value of retest reliability ranges from 0.83-1.00, and GIC can predict mortality (RR: 2.3, 95%CI: 1.7-3.1), but its adaptability is limited |
| 36 | Seattle Index of Co-morbidity（SIC） | SIC includes data on chronic diseases, smoking, and age to develop and validate a comorbidity index | Health care needs | The SIC scale had good reliability and showed good predictive power for mortality in the validation set (AUC = 0.71), but its adaptability is limited |
| 37 | The Index of Co-existent Disease（ICED） | Defined by the severity of the disease severity of the physical impairment category | Physiological function status | ICED has high inter-reliability and lacks adaptability and evaluation of its criterion validity |
| 38 | The Comorbidity Symptom Scale（CmSS） | The CmSS contains disease lists and quantitative symptom scores that reflect information routinely collected in clinical practice | Severity of the disease | CmSS scores correlated with activities of daily living, perceived health, and assessments of anxiety and depression (total instrument score *r* = 0.87 (*P*＜0.001),and the validity is good. But its adaptability is limited. |
| 39 | Elixhauser Index（EI） | For each disease, there are 30 binary indicators; No weighting system is used; Each indicator acts as an independent predictor | A chronic health condition | Ordinary least squares regression analysis found that the number of illnesses (0, 1, 2, 3 or more) predicted length of stay and hospitalization costs. Logistic regression analysis showed that the number of diseases can predict the proportion of deaths during hospitalization. |

**Continue to Supplementary Table 1**

| **Number** | **Evaluation tools** | **Tool characteristics** | **Evaluation dimension** | **Validity evaluation** |
| --- | --- | --- | --- | --- |
| 40 | Incalzi Index | The Incalzi index quantifies the prognostic weight of an individual disease and tests the predictive power of variables univariately related to the outcome through logistic regression. | Acute medical diseases of the aged | The weighted Kappa test assessed consistency, indicating good agreement (P < 0.001), and the age-comorbidities index was the second strongest predictor of in-hospital mortality, with strong adaptability |
| 41 | Standardized Comorbidity Measurement | Thirty-eight comorbidities ranked high in the ranked list were selected, and stepwise multiple regression was used to classify patient status into six levels | Physiological function status | The consistency of CI and w-CI was 0.896 and 0.997, respectively, and they were significantly correlated with Charlson index and its weighted version (Spearman's rank correlation coefficients ranged from 0.397 to 0.925). |
| 42 | Shwartz Comorbidity Scores | A stepwise regression model was used to compile comorbidity scores. | Acute and chronic physiological function status | The comorbidity scale has good reliability, validity and adaptability |
| 43 | McGee Comorbidity Score | Logistic regression model and proportional hazards model were used to explore the relationship between comorbidity index and prognosis | Disease burden | Both comorbidity scoring methods have good reliability and certain validity, but its adaptability is limited. McGee Comorbidity Score was different among different race-sex groups and significantly correlated with survival rate |
| 44 | Chronic Disease Score -Clark（CDS-Clark） | Based on regression models, parameters estimated for each drug are used to predict healthcare cost indicators | Chronic physical and mental dysfunction | CDS-Clark was able to explain 10 percent of the variation in total costs, 23 percent of outpatient costs, and 13 percent of primary care visits |
| 45 | four level Index of co-existent Disease（ICED） | Patients were ranked according to the severity of comorbid conditions and physical impairments, and the two subscales were condensed into the ICED index | Physiological function status | ICED scale has good reliability and validity, can effectively predict postoperative complications and long-term health outcomes of patients with total hip replacement, and has high adaptability |
| 46 | CCI adaptation Roman | Cumulative index of 17 health conditions based on original Charlson Comorbidity Index (CCI) weights | Chronic physiological function status | CCI is based on multiple independent comorbidities as predictors, which guarantees internal consistency and high content validity of the scale, but its adaptability is limited |
| 47 | The Duke Severity of Illness Checklis（DUSOI） | The DUSOI score is based on a composite assessment of four non-disease-specific parameters: severity of symptoms, complications, prognosis without treatment, and expected response to treatment | physiological function status | DUSOI shows good assessment validity in assessing overall severity |
| 48 | Chronic Disease Score（CDS） | CDS scores are evaluated based on their stability over time and their association with other indicators of health status | Chronic physiological health status | CDS scores correlated with physician ratings of physical illness severity (r = 0.57); It has good reliability and adaptability |
| 49 | Cumulative Illness Rating Scale-geriatric version（CIRS-G） | CIRS-G is the number of items reporting moderate to severe pathology to derive an overall severity score | Chronic physiological dysfunction | The CIRS(G) scale performed well in reliability and validity, and was highly adaptable. The test result of inter-rater consistency was 0.78 |
| 50 | Deyo adaptation Charlson | ICD-9-CM coding to identify all 19 Charlson comorbidity index conditions; Combining leukemia and lymphoma with other malignancies; Use the original CCI weights | Chronic physical health condition | The modified Charlson index has good reliability, validity and adaptability |

**Continue to Supplementary Table 1**

| **Number** | **Evaluation tools** | **Tool characteristics** | **Evaluation dimension** | **Validity evaluation** |
| --- | --- | --- | --- | --- |
| 51 | Ambulatory Care  Groups（ACG） | Clustering of diagnoses based on expected use of medical services | Chronic health condition | ACGs were able to predict 15% of the variability in doctor visits over the next year. After further factoring in age and gender, this predictive power increased to 20% |
| 52 | Charlson Index | The severity of each disease or condition is coded on a scale from 0 to 6 based on its 1-year relative risk of death, and a weighted index is calculated accordingly | Chronic physical health condition | As the comorbidity index gradually increases from level 0 to level 6, the 10-year mortality rate of patients also shows an increasing trend; the results of the chi-square test show that this relationship is statistically different（χ²=165，P<0.0001） |
| 53 | Kaplan-Feinstein Index | The Kaplan-Feinstein index has four levels | Severity of the disease | The comorbidities classification scale performed well in reliability, validity and adaptability. |
| 54 | Cumulative Illness Rating Scale（CIRS） | By summing the 13 items, a total pathology score was calculated to represent the injury of the individual as a whole. The ratings are assessed on a 5-point “severity” scale, ranging from “none” to “extremely serious” | Chronic physiological dysfunction | Kendall's W values were 0.83, 0.85, 0.86 and 0.91 (P < 0.01), respectively, indicating good validity. but its adaptability is limited. |

**Supplementary Table 2** List of Diseases in multiple chronic disease assessment tools

| **Number** | **Evaluation tools** | **Amount（Disease/health status）** | **Disease/health status** |
| --- | --- | --- | --- |
| 1 | The Multimorbidity Index | 12 | Sensory disorders, memory-related disorders, asthma, mental illness, stroke, heart disease, liver disease, chronic lung disease, cancer, diabetes, high blood pressure, stomach and other digestive disorders |
| 2 | The Simpliﬁed Comorbidity Index（SCI） | 8 | Acute myeloid leukemia, chronic myeloid leukemia, acute lymphoblastic leukemia, other leukemias, multiple myeloma, myeloproliferative neoplasms, lymphoma, myelodysplastic syndrome |
| 3 | The Chronic Lymphocytic Leukemia Comorbidity Index (CLL-CI) | 5 | Diabetes, obesity, upper gastrointestinal disease, vascular disease, immunoglobulin heavy chain variable region status |
| 4 | The Metabolic Health Index（MHI） | 18 | Diabetes, dyslipidemia, obesity, gastric bypass or sleeve gastrectomy, hemoglobin value, hematocrit value, mean corpuscular volume value, platelet value, sodium value, calcium value, ferritin value, folic acid value, albumin value, C Amino acid transaminase value, glucose value, total cholesterol value, high-density lipoprotein value, cholesterol value |
| 5 | Quality of Life Disease Impact Scale（QDIS) | 35 | Hypertension, myocardial infarction, angina, congestive heart failure, diabetes, stroke, cancer, asthma, chronic obstructive pulmonary disease, chronic kidney disease, rheumatoid arthritis, osteoarthritis, osteoporosis, ulcers, liver disease, Irritable bowel syndrome, obesity, AIDS/HIV infection, anemia, depression, chronic fatigue syndrome, fibromyalgia, migraines, prostate disease, erectile dysfunction, hypothyroidism, chronic allergies, seasonal allergies, chronic back pain , vision problems, hearing problems, osteoporosis, limited use of limbs, foot/ankle problems, hip/knee problems |
| 6 | Whitney Comorbidity Index（WCI） | 27 | Any malignancy, blood loss, deficiency anemia, cerebrovascular disease, chronic lung disease, dementia, complex diabetes, simple diabetes, liver disease, myocardial infarction, kidney disease, metastatic cancer, cardiac arrhythmia, congestive heart failure, depression, epilepsy, Fluid and electrolyte disorders, high blood pressure, hypothyroidism, neurological disorders, rheumatoid arthritis, other inflammatory polyarticular diseases, fragile bones, difficulty swallowing, gastrointestinal problems, intellectual disability, neurogenic bowel or bladder disease, bone and joints inflammation and related diseases, pneumonia |
| 7 | The cardio-Canary Comorbidity Project | 5 | Hypertension, dyslipidemia, diabetes, coronary artery disease, stroke/transient ischemic attack |
| 8 | Subjective Global Assessment–Dialysis Malnutrition Score（SGA-DMS） | 7 | Weight changes, dietary intake, gastrointestinal symptoms, nutrition-related impairments, comorbidities, reduced fat storage/ reduced subcutaneous fat, muscle atrophy |
| 9 | Multimorbidity Weighted Index（MWI） | 17 | History of knee replacement surgery, dementia, delirium, congestive heart failure, lung disease, stroke, history of hip replacement surgery, arthritis, connective tissue disease, diabetes, angina, myocardial infarction, irregular heartbeat, cancer(except skin cancer), other heart disease problems, glaucoma, high blood pressure |
| 10 | M3 index | 61 | AIDS, alcoholism, iron deficiency anemia, anxiety with behavioral disorders, aortic aneurysm/other aneurysms, skeletal abnormalities, inflammatory bowel disease, breast cancer, cardiac arrhythmia, heart valve disease, cerebrovascular disease, chronic lung disease, chronic renal function hypotension, coagulopathy/other blood disorders, colorectal cancer, congestive heart failure, connective tissue disease, dementia, complex diabetes, uncomplicated diabetes, substance abuse, endocrine disorders, epilepsy, long-term eye problems, gastrointestinal ulcer disease/ Other upper gastrointestinal diseases, gynecological cancers, hepatitis/chronic viral hepatitis, simple hypertension, immune system disorders, hearing problems, joint/spine diseases, liver disease (moderate/severe), lung cancer, lymphoma/leukemia, severe mental illness disorders, malignant melanoma, malnutrition/other nutritional disorders, mental and behavioral disorders caused by brain injury, mental retardation, metabolic disorders, metastatic cancer, peripheral nerve disorders, myocardial infarction, obesity, simple osteoporosis, Other cancers, other neurological disorders (excluding epilepsy), paralysis, peripheral vascular disease, prostate cancer, pulmonary circulatory disorders, sleep disorders, upper gastrointestinal cancer, chronic urinary tract problems, venous insufficiency, angina, other heart disease, chronic infections , intestinal diseases, pancreatitis, tuberculosis |
| 11 | The revised Myeloma Comorbidity Index（R-MCI) | 13 | Kidney disease, lung disease, Karnofsky functional status score, cardiac disease, liver disease, gastrointestinal disease, disability, frailty, infection, thromboembolic events, peripheral neuropathy, pain, secondary malignancy |
| 12 | Self-Reported Chronic Disease Assessment Questionnaire | 33 | Hypertension, depression, anxiety, chronic musculoskeletal disorders, arthritis/rheumatoid arthritis, osteoporosis, asthma, chronic obstructive pulmonary disease, chronic bronchitis, angina, myocardial infarction, atrial fibrillation, lower extremity circulation Heart failure, valvular heart disease, stroke/transient ischemic attack, gastric reflux, heartburn, gastric ulcer, irritable bowel syndrome, Crohn's disease, ulcerative colitis, diverticular disease, chronic hepatitis, Diabetes, thyroid disease, cancer (including melanoma but not other skin cancers), kidney failure, chronic urinary disease, dementia, Alzheimer's disease, hyperlipidemia, obesity |

**Continue to Supplementary Table 2**

| **Number** | **Evaluation tools** | **Amount（Disease/health status）** | **Disease/health status** |
| --- | --- | --- | --- |
| 13 | The ADHD Concomitant Difﬁculties Scale（ADHD-CDS） | 13 | Self-esteem level, emotional self-regulation ability, writing level, manual ability, problem-solving ability, time management ability, time series processing ability, extreme level, quality of life, school diary writing ability, academic ability, reading comprehension ability, mathematical ability |
| 14 | Elixhauser-based Comorbidity Summary Measure | 30 | Congestive heart failure, arrhythmias, valvular heart disease, pulmonary circulation disorders, peripheral vascular disease, hypertension, paralysis, other neurological diseases, chronic lung disease, complex diabetes, simple diabetes, hypothyroidism, renal failure, liver disease, digestive Ulcerative disease, AIDS/HIV infection, lymphoma, metastatic cancer, solid tumors without metastasis, rheumatoid arthritis, coagulopathy, obesity, weight loss, water and electrolyte imbalance, blood loss anemia, iron deficiency anemia, Alcohol abuse, drug abuse, mental illness, depression |
| 15 | the Pharmacy-Based Disease Indicator （PBDI） | 20 | AIDS, malignant tumors, ischemic heart disease, cerebrovascular disease, tuberculosis, chronic obstructive pulmonary disease, diabetes, asthma, liver cirrhosis, Alzheimer's disease/other dementias, nephritis/other kidney diseases, epilepsy, hypertension Sexual heart disease, rheumatic heart disease, skin diseases, peptic ulcer disease, benign prostatic hypertrophy, hepatitis B, hepatitis C, Parkinson's disease |
| 16 | EI adaptation van Walraven | 30 | Congestive heart failure, arrhythmia, valvular heart disease, pulmonary circulatory disorders, peripheral vascular disease, hypertension, paralysis, neurodegenerative disease, chronic lung disease, simple diabetes, complex diabetes, hypothyroidism, renal failure, liver Disease, peptic ulcer disease, AIDS/HIV infection, lymphoma, metastatic cancer, non-metastatic solid tumors, rheumatoid arthritis/collagen vascular disease, coagulopathies, obesity, weight loss, fluid/electrolyte imbalance, hemorrhagic Anemia, deficiency anemia, alcohol abuse, drug abuse, psychosis, depression |
| 17 | Weighted Multimorbidity Indexes | 19 | Alzheimer's Disease, Depression, Anxiety, Arthritis, Asthma, Bronchitis/Emphysema, Chest Pain, Diabetes, Serious Injury from Falls, Fractures from Falls, Medical Care from Falls, Heart Disease, High Blood Pressure, Low Iron , other cancers, osteoporosis, skin cancer, stroke, urinary incontinence |
| 18 | A physiologic Index of Comorbidity | 7 | Arthritis, cerebrovascular disease, chronic obstructive pulmonary disease, coronary heart disease, depression, diabetes, kidney disease |
| 19 | CCI adaptation Klabunde | 16 | Chronic lung disease, diabetes, congestive heart failure, cerebrovascular disease, peripheral vascular disease, myocardial infarction, rheumatism, acute myocardial infarction, moderate/severe renal disease, complex diabetes, dementia, ulcer disease, paralysis, mild liver disease, Moderate/severe liver disease, HIV |
| 20 | Medication-Based Disease Burden Index（MDBI） | 20 | Alzheimer's disease/other dementias, asthma, benign prostatic hypertrophy, cerebrovascular disease, cirrhosis, chronic obstructive pulmonary disease, diabetes, epilepsy, hepatitis B, hepatitis C, AIDS/HIV infection, hypertension and heart disease, Ischemic heart disease, malignant tumors, nephritis/other kidney diseases, Parkinson's disease, peptic ulcer disease, rheumatic heart disease, skin diseases, tuberculosis |
| 21 | Functional Comorbidity  Index（FCI） | 25 | Anemia, Angina, Anxiety, Arthritis, Asthma, Back Pain, Bowel Disease, Cancer, Heart Failure, COPD, Depression, Dementia, Diabetes, Hearing Problems, High Blood Pressure, Kidney Disease, liver disease, Myocardial Infarction, Migraines, neurological disorders, osteoporosis, peripheral vascular disease, stroke/transient ischemic attack, upper gastrointestinal disorders, vision problems |
| 22 | The DVA PCT Multimorbidity Questionnaire | 26 | Angina/chest pain, arthritis, rheumatism, high blood pressure, cancer, chronic allergies/sinus problems, chronic lung disease, chronic pain, depression, dermatitis/other chronic skin conditions, diabetes, digestive problems, fainting, forgetfulness, gallbladder disease, hearing Problems, heart disease, heart bypass, heart failure, kidney disease, liver disease, muscle weakness/spasms, sciatica/back or spine problems, stroke, urinary tract problems, vision problems |
| 23 | Subjective Assessments of Comorbidity | 23 | Angina/coronary artery disease, asthma, back pain, chronic bronchitis/COPD, cancer, high cholesterol, colon problems, heart failure, diabetes, hearing problems, high blood pressure, kidney disease, neurological conditions, osteoarthritis, Osteoporosis, overweight, poor circulation, rheumatic diseases, rheumatoid arthritis, stomach problems, stroke, thyroid disease, vision problems |
| 24 | New ICD-10 version of Charlson Comorbidity Index | 12 | Heart disease, peripheral vascular disease, cerebrovascular disease, dementia, lung disease, connective tissue disease, digestive system disease, diabetes, hemiplegia, kidney disease, cancer, AIDS |
| 25 | The Self-Administered Comorbidity Questionnaire（SCQ） | 15 | Anemia/other blood disorders, back pain, cancer, depression, diabetes, heart disease, high blood pressure, kidney disease, liver disease, lung disease, other health problems, osteoarthritis, degenerative arthritis, rheumatoid arthritis, ulcers diseases/stomach problems |
| 26 | CDS adaptation RxRisk | 57 | Anxiety/Stress, Asthma, Bipolar Disorder, Heart Disease, Coronary/Peripheral Vascular Disease, Cystic Fibrosis, Depression, Diabetes, Epilepsy, End-Stage Renal Disease, Acid Disorders, Glaucoma, Gout, Heart Disease/Hypertension , AIDS, hyperlipidemia, irritable bowel syndrome, liver failure, malignant tumors, Parkinson's disease, mental illness, kidney disease, rheumatoid arthritis, thyroid disease, organ transplantation, tuberculosis, acne, allergic rhinitis, Amino acid disorders, attention deficit disorder, central venous catheter, congenital adrenal insufficiency, eczema, growth hormone deficiency, immunodeficiency, liver disease, pain, inflammation, pituitary hormones, sickle cell anemia, steroid-dependent disease Combine the same health conditions for adults and children) |

**Continue to Supplementary Table 2**

| **Number** | **Evaluation tools** | **Amount（Disease/health status）** | **Disease/health status** |
| --- | --- | --- | --- |
| 27 | The CMS Hierarchical Condition Categories Model（CMS-HCC） | 70 | AIDS/HIV infection, sepsis/shock, opportunistic infection, metastatic cancer/acute leukemia, lung cancer/upper gastrointestinal tract cancer, lymphoma/head and neck cancer/brain cancer, breast cancer/prostate cancer/colorectal cancer, diabetes with renal or peripheral Circulatory manifestations, diabetes with neurologic changes, diabetes with acute complications, diabetes with vision changes, uncomplicated diabetes, protein-calorie malnutrition, end-stage liver disease, cirrhosis, chronic hepatitis, intestinal obstruction/perforation, pancreatic disease, inflammatory Enteropathy, bone/joint/muscle infection/necrosis, rheumatoid arthritis/inflammatory connective tissue disease, severe blood disease, immune disease, drug/alcoholic psychosis, drug/alcohol dependence, schizophrenia, major depression/ Bipolar/paranoid disorder, quadriplegia, paraplegia, spinal cord disease/injury, muscular dystrophy, polyneuropathy, multiple sclerosis, Parkinson's/Huntington's disease, epilepsy/convulsions, coma/anoxic brain injury, ventilator Dependence/tracheostomy state, respiratory arrest, cardiopulmonary failure/shock, congestive heart failure, acute myocardial infarction, unstable angina pectoris/other acute ischemic heart disease, angina pectoris/elderly myocardial infarction, specific arrhythmia, brain Hemorrhage, ischemic or nonspecific stroke, hemiplegia/hemiparesis, cerebral palsy and other paralytic syndromes, vascular disease with complications, vascular disease, Cystic fibrosis, chronic obstructive pulmonary disease, aspiration and specific bacterial pneumonia, pneumococcal pneumonia/empyema/pulmonary abscess, proliferative diabetic retinopathy with vitreous hemorrhage, dialysis status, renal failure, nephritis, skin pressure Injuries, chronic skin ulcers, extensive third-degree burns, severe head injuries, severe head injuries, vertebral fractures without spinal cord injury, hip fractures/dislocations, traumatic amputations, medical care and major complications of trauma, major organ transplants Condition, with artificial opening for eating or excreting, amputation status/complications of amputation |
| 28 | Geriatric Index of Comorbidity | 7 | Anemia, heart disease, musculoskeletal disorders, Parkinson's disease, peripheral vascular disease, kidney disease, stroke |
| 29 | Seattle Index of Comorbidity（SIC） | 25 | Angina, other heart disease, coronary artery bypass surgery, percutaneous coronary angioplasty, coronary artery disease, myocardial infarction, congestive heart failure, arthritis, osteoporosis, cancer, depression, diabetes, substance abuse, benign prostate Hyperplasia, heartburn, hypertension, AIDS, liver disease, lung disease, post-traumatic stress disorder, renal insufficiency, epilepsy, stroke, thyroid disease, ulcer disease |
| 30 | The Index of Coexistent Disease（ICED） | 30 | Active phase of symptoms requiring continued treatment, acute conditions requiring prompt treatment, ischemic heart disease, congestive heart failure, arrhythmia, other heart diseases, hypertension, cerebrovascular disease, peripheral vascular disease, diabetes, respiratory disease, malignancy Tumors, liver diseases, gastrointestinal diseases, neurological diseases, arthritis, blood diseases, AIDS, period of anticoagulant medication use, circulatory dysfunction, dyspnea, neurological dysfunction, psychological dysfunction, difficulty urinating, difficulty defecating, eating Impairments, walking impairments, vision problems, hearing problems, speech impairments |
| 31 | The Comorbidity Symptom Scale（CmSS） | 23 | Angina, anxiety/depression, arthritis/osteoporosis, dyspnea secondary to cardiovascular causes, dyspnea/wheezing secondary to respiratory causes, cough secondary to chronic obstructive pulmonary disease/asthma/ Coughing phlegm, cerebrovascular disease, constipation, diabetes, diarrhea, epilepsy, hearing problems, Parkinson's disease, pain, peripheral vascular disease, medication side effects, skin disorders, falls/fainting, upper gastrointestinal symptoms, urinary tract problems, vision problems , walking and activity functional status, other health problems |
| 32 | Elixhauser Index | 30 | Heart failure, myocardial infarction, atrial fibrillation/atrial flutter, heart valve disease, pulmonary circulation disease, chronic obstructive pulmonary disease, respiratory failure, asthma, complex diabetes, simple diabetes, uncontrolled diabetes, prediabetes, acute myocardial infarction, Chronic heart failure, peripheral vascular disease, cerebrovascular disease, dementia, chronic kidney disease, anemia, lymphoma, hematological malignancies, multiple myeloma, leukemia, lung cancer, malignant tumors, metastatic cancer, AIDS, liver disease, rheumatism , ulcer disease |
| 33 | Incalzi Index | 48 | Acute leukemia, acute pancreatitis, anemia, aplastic anemia (blood transfusion interval ＞20 days), aplastic anemia (blood transfusion interval ≤20 days), chronic active hepatitis/cirrhosis, chronic lymphocytic leukemia (AB stage) , chronic lymphocytic leukemia (stage C), chronic myelogenous leukemia (early stage), chronic myelogenous leukemia (cellular crisis), chronic obstructive pulmonary disease, chronic respiratory failure (stage 1, hypoxemia), chronic respiratory failure (stage 2-3, hypoxemia with hypercarbemia), ulcerative colitis, collagenosis with organ damage, congestive heart failure (NYHAI-II), congestive heart failure (NYHA III- IV), stage 3 pressure injury, simple dementia, dementia with complications, diabetes, diabetes with organ damage, Hodgkin's disease (stage I-II), Hodgkin's disease (stage III), hypertension, Hyperthyroidism, infective endocarditis, ischemic stroke, hepatic steatosis, mild liver disease, meningitis/encephalitis, mild renal failure, multiple myeloma (stage I), multiple myeloma (Phase III), multiple sclerosis, myasthenia gravis, metastatic tumor disease, non-Hodgkin lymphoma, parenchymal cerebral hemorrhage, Parkinson's disease with comorbidities, peptic ulcer disease, peripheral arterial disease, peritoneal cancer, Pulmonary embolism, sepsis, severe renal failure, transient ischemic attack, tumors |
| 34 | Standardized Comorbidity Measures | 38 | Hypertension, atrial fibrillation, premature ventricular contractions, angina pectoris, myocardial infarction, valvular heart disease, heart failure, abnormal electrocardiogram, pneumonia, chronic respiratory failure, shoulder joint pain, other pain, hyperlipidemia, obesity, weight loss, diabetes, liver disease Functional impairment, electrolyte abnormalities, pancreatitis, peptic ulcer disease, gastritis, stones, constipation, hemorrhoids, depression, dementia, epilepsy, vision problems, hearing problems, neurogenic bladder, urinary tract infection, urinary tract stones, anemia, other Infections, tumors, skin diseases, eczema, dental diseases |
| 35 | Shwartz Comorbidity Scores | 8 | Acute and chronic bronchitis/asthma, acute myocardial infarction, cerebrovascular disease, coronary artery disease without acute myocardial infarction, hip/femoral fracture, prostate disease, respiratory malignancy, spine/back disease |
| 36 | McGee Comorbidity Scores | 4 | Coronary heart disease, diabetes, hypertension, stroke |

**Continue to Supplementary Table 2**

| **Number** | **Evaluation tools** | **Amount（Disease/health status）** | **Disease/health status** |
| --- | --- | --- | --- |
| 37 | Chronic Disease Score -Clark（CDS-Clark） | 30 | Coronary/Peripheral Vascular Disease, Epilepsy, Hypertension, HIV, Tuberculosis, Rheumatoid Arthritis, High Cholesterol, Malignancy, Chronic Kidney Disease, Heart Disease, Diabetes, Glaucoma, Cystic Fibrosis, Kidney Failure, Liver Failure, Ulcers, Post-Transplant, Respiratory Disease, Asthma, Thyroid Disease, Gout, Crohn's Disease/Intestinal Inflammation, Pain, Inflammation, Depression, Psychosis, Mania, Anxiety/Stress, Pain, Parkinson's Disease |
| 38 | Four level Index of co-existent Disease（ICED） | 13 | Cerebrovascular disease, heart failure, diabetes, liver disease, gastrointestinal disease, hypertension, ischemic heart disease, malignant tumors (excluding basal cell carcinoma of the skin), organic heart disease, peripheral vascular disease, primary cardiac rhythm Abnormalities and conduction block problems, kidney disease, respiratory disease |
| 39 | CCI adaptation  Roman | 17 | Myocardial infarction, heart failure, peripheral vascular disease, cerebrovascular disease, dementia, chronic lung disease, rheumatic disease, peptic ulcer disease, mild liver disease, mild/moderate diabetes, diabetes with chronic complications, hemiplegia/paraplegia, kidney disease, any malignancy, moderate/severe liver disease, metastatic solid tumors, AIDS |
| 40 | The Duke Severity of Illness Checklist（DUSOI） | 21 | Sprains/Strains, Neurological Disorders, Headaches, Acute Bronchitis, Obesity, Low Back Pain, Acute/Chronic Sinusitis, Chronic Ischemic Heart Disease, Anxiety, Stomach Disorders, Diabetes, Alcohol Abuse, Bruises/Contusions, Depression, Osteoarthritis, acute upper respiratory tract infection, smoking, hypertension, vaginitis, lipid disorders, menopausal syndrome |
| 41 | Chronic Disease Score（CDS） | 19 | Acne, rheumatism, asthma, rhinitis, cancer, diabetes, epilepsy, glaucoma, gout, hyperuricemia, heart disease, high cholesterol, high blood pressure, migraines, Parkinson's disease, respiratory diseases, rheumatoid arthritis, Tuberculosis, ulcer disease |
| 42 | =Cumulative Illness Rating Scale-geriatric version（CIRS-G） | 14 | Heart disease, vascular disease, hematopoiesis, respiratory disease, otolaryngology disease, upper gastrointestinal disease, lower gastrointestinal disease, liver disease, kidney disease, reproductive/urinary disease, musculoskeletal/skin disease, neurological disease, endocrine/metabolic system diseases/breast diseases, mental diseases |
| 43 | Deyo adaptation Charlson | 17 | Myocardial infarction, heart failure, peripheral vascular disease, cerebrovascular disease, dementia, chronic lung disease, rheumatism, peptic ulcer, mild liver disease, moderate/severe liver disease, diabetes, complex diabetes, hemiplegia/paraplegia, kidney disease, any malignancy Neoplasms, Metastatic Solid Tumors, HIV |
| 44 | Ambulatory Care  Groups（ACG） | 51 | 51 different ACG categories are divided according to disease type, age and gender. Category characteristics are not specified in the article. |
| 45 | Charlson Index | 30 | Angina pectoris, arrhythmia, valvular heart disease, myocardial infarction, congestive heart failure, hypertension, peripheral vascular disease, cerebrovascular disease, mild pulmonary disease, moderate to severe pulmonary disease, other neurological pulmonary diseases, dementia, hemiplegia / Paraplegia, other endocrine system diseases, diabetes, end-stage diabetes, mild renal insufficiency, moderate/severe renal insufficiency, mild hepatic insufficiency, moderate/severe hepatic insufficiency, gastrointestinal bleeding, enteritis, peptic ulcer, Tumors, lymphomas, leukemias, AIDS, metastatic cancer, rheumatism, coagulation disorders |
| 46 | Kaplan-Feinstein index | 12 | Hypertension, cardiac disease, neuropsychiatric disease, respiratory disease, kidney disease, liver disease, gastrointestinal disease, peripheral vascular disease, malignancy, motor dysfunction, alcohol abuse, other health problems |
| 47 | Cumulative Illness Rating Scale（CIRS） | 13 | Cardiac parenchymal diseases, cardiovascular diseases, respiratory diseases, otolaryngology diseases, upper gastrointestinal diseases, lower gastrointestinal diseases, liver diseases, kidney diseases, other urogenital diseases, muscle/skeletal/skin diseases, neurological diseases, mental and psychological diseases, endocrine and metabolic diseases |
| 48 | Multisource Comorbidity Score（MCS） | 34 | Metastatic cancer, alcoholism, non-metastatic cancer, tuberculosis, psychosis, liver disease, use of anti-anxiety medications, weight loss, dementia, malignancy medications, Parkinson's disease, lymphoma, hemiplegia, coagulation disorders, fluid disorders, electrolyte disorders, Acid-base disorders, kidney disease, renal dialysis, heart failure, other neurological diseases, rheumatoid arthritis, anemia, cerebrovascular disease, diabetes, vascular disease, gout, epilepsy, chronic lung disease, peptic ulcer, acute Myocardial infarction, coronary artery disease, peripheral vascular disease, heart valve disease, cardiac arrhythmias, obesity, hypothyroidism |
| 49 | Tonelli Administrative algorithms | 30 | Alcohol abuse, asthma, atrial fibrillation, lymphoma, metastatic cancer, non-metastatic cancer, chronic heart failure, chronic kidney disease, chronic pain, chronic lung disease, chronic viral hepatitis B, cirrhosis, hepatic encephalopathy, dementia , depression, diabetes, epilepsy, hypertension, hypothyroidism, inflammatory bowel disease, irritable bowel syndrome, multiple sclerosis, myocardial infarction, Parkinson's disease, peptic ulcer disease, peripheral vascular disease, psoriasis, Rheumatoid arthritis, schizophrenia, severe constipation, stroke, transient ischemic attack |
| 50 | Modified Version of the Cambridge Multimorbidity Score | 17 | Hypertension、Asthma、Diabetes、Thyroid disorders、Chronic kidney disease、Atrial fibrillation、Cancer、Chronic obstructive pulmonary disease、Alcohol problems、Stroke and transient ischemic attack、Constipation、Chronic sinusitis、Coronary heart disease、Heart failure、Dementia、Epilepsy、Schizophrenia or bipolar disorder |
| 51 | Cambridge Multimorbidity Score | 37 | Diabetes、Hypertension、Chronic kidney disease、Chronic obstructive pulmonary disease、Asthma、Atrial fibrillation、Schizophrenia or bipolar disease、Epilepsy、Parkinsonism、Dementia、Coronary heart disease、Congestive heart failure、Peripheral vascular disease、Ischaemic stroke、Transient ischaemic attack、Alcohol problems、Chronic liver disease and viral hepatitis、Peptic ulcer disease、Inflammatory bowel disease、Psoriasis or eczema、Anxiety or depression、Blindness and low vision、Deafness、Disorders of the prostate、Osteoporosis、Rheumatoid arthritis/seropositive polyarthritis、Other degenerative or inflammatory joint disease、Connective tissue disease、Benign prostatic hypertrophy、Gout or other crystal arthropathy、Learning disability、Irritable bowel syndrome、Diverticular disease of the intestine、Anorexia or bulimia、Parkinson's disease、Multiple sclerosis、Chronic sinusitis |

**Continue to Supplementary Table 2**

| **Number** | **Evaluation tools** | **Amount（Disease/health status）** | **Disease/health status** |
| --- | --- | --- | --- |
| 52 | Chinese multimorbidity-weighted index (CMWI) | 14 | Hypertension、Diabetes or high blood sugar、Cancer or malignant tumour、Chronic lung diseases、Liver disease、Heart disease、Stroke、Kidney disease、Stomach or other digestive diseases、Emotional, nervous or psychiatric problems、Memory-related diseases、Arthritis or rheumatism、Asthma、Glaucoma |
| 53 | Multimorbidity indices with  individual diseases(MI)，Multimorbidity Index incorporating Disease Combinations (MIDC) | 13 | Hypertension、Diabetes、Cerebrovascular disease、Heart disease、Cancer、Lung disease、Parkinson’s disease、Arthritis、Tooth loss、Cognitive impairment、Depressive symptoms、Sensory impairment、Bedridden status |
| 54 | multimorbidity-weighted index (MWI)，Multimorbidity-weighted index ICD-coded conditions (MICD) | 17 | Hypertension、Osteoarthritis、Other musculoskeletal conditions、Coronary artery disease、Back pain、Diabetes、Anemia、Fluid, electrolyte and acid-base disorders、Chronic obstructive pulmonary disease、ALS、Parkinson disease、Paralysis、Limb amputation、Hip fracture、Lung cancer、Congestive heart failure、Dementia |

**Supplementary Table 3**

Risk of bias of the RCTs (n=3)

| **Study** | **A1** | **A2** | **A3** | **A4** | **A5** | **A6** | **A7** | **Level of evidence** |
| --- | --- | --- | --- | --- | --- | --- | --- | --- |
| Miskulin DC 2001 | Unclear risk of bias | Unclear risk of bias | Low risk of bias | Low risk of bias | Low risk of bias | Low risk of bias | Low risk of bias | B |
| Miller MD 1992 | Unclear risk of bias | Unclear risk of bias | Low risk of bias | Low risk of bias | Unclear risk of bias | Low risk of bias | Low risk of bias | B |
| Byles JE 2005 | Low risk of bias | Unclear risk of bias | Low risk of bias | High risk of bias | Low risk of bias | Low risk of bias | Unclear risk of bias | B |

**Note: A1**=Random sequence generation (selection bias); **A2**=Allocation concealment (selection bias); **A3**=Blinding of participants and personnel (performance bias); **A4**=Blinding of outcome assessment (detection bias); **A5**=Incomplete outcome data (attrition bias); **A6**=Selective reporting (reporting bias); **A7**=Other bias

Reference:

1. Sterne JAC, Savović J, Page MJ, et al. RoB 2: a revised tool for assessing risk of bias in randomised trials. *BMJ*. 2019;366:l4898.. doi:10.1136/bmj.l4898

**Supplementary Table 4**

Risk of bias of the cohort studies (n=28)

| **Study** | **C1** | **C2** | **C3** | **C4** | **C5** | **C6** | **C7** | **C8** | **Quality Scores** |
| --- | --- | --- | --- | --- | --- | --- | --- | --- | --- |
| Xu HW 2024 | 1 | 1 | 1 | 0 | 2 | 1 | 1 | 1 | 8 |
| Shouval R 2022 | 1 | 1 | 1 | 1 | 1 | 1 | 1 | 1 | 8 |
| Rotbain EC 2022 | 1 | 1 | 1 | 1 | 1 | 1 | 1 | 0 | 7 |
| Gensen C 2022 | 1 | 1 | 1 | 1 | 2 | 1 | 1 | 1 | 9 |
| Whitney DG 2021 | 1 | 1 | 1 | 1 | 1 | 1 | 0 | 0 | 6 |
| Berman AN 2021 | 1 | 1 | 1 | 1 | 1 | 1 | 1 | 1 | 8 |
| Spatola L 2019 | 1 | 1 | 1 | 1 | 1 | 1 | 1 | 1 | 8 |
| Stanley J 2017 | 1 | 1 | 1 | 1 | 2 | 1 | 1 | 1 | 9 |
| Engelhardt M 2017 | 1 | 1 | 1 | 1 | 2 | 1 | 1 | 1 | 9 |
| Fenollar-Cortés J 2016 | 1 | 0 | 1 | 1 | 2 | 1 | 1 | 1 | 8 |
| Thompson NR 2015 | 1 | 1 | 1 | 1 | 1 | 1 | 0 | 0 | 6 |
| Dong YH | 1 | 1 | 1 | 1 | 1 | 1 | 1 | 1 | 8 |
| Klabunde CN 2007 | 1 | 1 | 1 | 1 | 1 | 1 | 1 | 1 | 8 |
| Sundararajan V 2004 | 1 | 1 | 1 | 1 | 1 | 1 | 1 | 1 | 8 |
| Fishman PA 2003 | 1 | 1 | 1 | 1 | 1 | 1 | 0 | 0 | 6 |
| Fan VS 2002 | 1 | 1 | 0 | 1 | 1 | 1 | 1 | 1 | 7 |
| Crabtree HL 2000 | 1 | 1 | 1 | 1 | 1 | 1 | 0 | 0 | 6 |
| Elixhauser A 1998 | 1 | 1 | 1 | 1 | 1 | 1 | 0 | 0 | 6 |
| Incalzi RA 1997 | 0 | 1 | 1 | 1 | 1 | 1 | 0 | 0 | 5 |
| McGee D 1996 | 1 | 1 | 1 | 1 | 2 | 1 | 1 | 0 | 8 |
| Charlson ME 1987 | 1 | 1 | 1 | 1 | 1 | 1 | 1 | 1 | 8 |
| Kaplan MH 1974 | 1 | 1 | 1 | 1 | 1 | 1 | 1 | 1 | 8 |
| Corrao G 2017 | 1 | 1 | 1 | 0 | 2 | 1 | 1 | 1 | 8 |
| Wei MY 2021 | 1 | 0 | 0 | 1 | 2 | 1 | 1 | 1 | 7 |
| Luo Y 2022 | 1 | 0 | 1 | 1 | 2 | 1 | 1 | 1 | 8 |
| Hu WH 2022 | 1 | 0 | 1 | 1 | 2 | 1 | 1 | 1 | 8 |
| Kar D 2024 | 1 | 0 | 1 | 1 | 2 | 1 | 1 | 1 | 8 |
| H. Harrison 2024 | 1 | 0 | 1 | 1 | 2 | 1 | 1 | 2 | 9 |

**C1=**Representativeness of the exposed cohort; **C2**=Selection of the nonexposed cohort; **C3**=Ascertainment of exposure; **C4**=Demonstration that outcome of interest was not present at start of study; **C5**=Comparability of cohorts on the basis of the design or analysis ; **C6**=Assessment of outcome; **C7**=Was follow up long enough for outcomes to occur ; **C8**=Adequacy of follow up of cohorts

Reference:

1.Stang A. Critical evaluation of the Newcastle-Ottawa scale for the assessment of the quality of nonrandomized studies in meta-analyses. *Eur J Epidemiol*. 2010;25(9):603-605. doi:10.1007/s10654-010-9491-z

**Supplementary Table 5**

Risk of bias of the Cross-sectional studies (n=15)

| **Study** | **S1** | **S2** | **S3** | **S4** | **S5** | **S6** | **S7** | **S8** | **Quality grade** |
| --- | --- | --- | --- | --- | --- | --- | --- | --- | --- |
| McEntee ML 2022 | Yes | Yes | No | No | Yes | Yes | unclear | Yes | B |
| Wei MY 2018 | Yes | Yes | Yes | Yes | unclear | unclear | Yes | Yes | B |
| van Walraven C 2009 | Yes | Yes | Yes | Yes | Yes | Yes | Yes | Yes | A |
| Tooth L 2008 | Yes | Yes | unclear | unclear | Yes | Yes | Yes | Yes | B |
| Newman AB 2008 | Yes | Yes | Yes | Yes | Yes | unclear | Yes | Yes | B |
| George J 2006 | Yes | Yes | Yes | Yes | Yes | Yes | Yes | Yes | A |
| Groll DL 2005 | Yes | Yes | No | No | Yes | unclear | unclear | Yes | B |
| Bayliss EA 2005 | Yes | Yes | Yes | Yes | Yes | unclear | No | Yes | B |
| Sangha O 2003 | unclear | Yes | Yes | Yes | Yes | unclear | Yes | Yes | B |
| Rozzini R 2002 | Yes | Yes | Yes | Yes | Yes | Yes | Yes | Yes | A |
| Shwartz M 1996 | Yes | Yes | unclear | Yes | unclear | Yes | Yes | Yes | B |
| Parkerson GR Jr 1993 | unclear | Yes | Yes | Yes | unclear | unclear | Yes | Yes | B |
| Deyo RA 1992 | Yes | Yes | unclear | Yes | unclear | Yes | Yes | Yes | B |
| Tonelli M 2015 | Yes | Yes | Yes | Yes | Yes | Yes | Yes | Yes | A |
| Von Korff M 1992 | unclear | Yes | unclear | Yes | Yes | Yes | Yes | Yes | B |

**S1=**Clearly defined inclusion criteria for study subjects? **S2**=A detailed description of the research object and the research site? **S3**=Use effective and reliable methods to measure exposure factors? **S4**=Use objective, standardized methods to measure health problems? **S5**=Have confounding factors been identified? **S6**=Measures taken to control confounding factors? **S7**=Use effective and credible methods to measure outcome indicators? **S8**=Is the data analysis method appropriate?

**Reference:**

1. Porritt K, Gomersall J, Lockwood C. JBI's Systematic Reviews: Study selection and critical appraisal. *Am J Nurs*. 2014;114(6):47-52. doi:10.1097/01.NAJ.0000450430.97383.64

**Supplementary Table 6**

The APPRAISE-AI Tool to Assess Quality of AI Studies in Medicine (n=8)

| **Study** | **Title** | **Introduction** | **Methods** | **Results** | **Discussion** | **Other information** | **Score** |
| --- | --- | --- | --- | --- | --- | --- | --- |
| Fortin M 2017 | 1 | 2 | 16 | 4 | 2 | 2 | 27 |
| Pope GC 2004 | 1 | 2 | 28 | 13 | 3 | 1 | 48 |
| Liu M | 1 | 2 | 32 | 11 | 3 | 2 | 51 |
| Clark DO 1995 | 0 | 2 | 19 | 9 | 3 | 1 | 34 |
| Greenfield S 1993 | 0 | 2 | 31 | 10 | 5 | 1 | 49 |
| Romano PS 1993 | 0 | 2 | 16 | 8 | 3 | 2 | 31 |
| Weiner JP 1991 | 1 | 2 | 24 | 12 | 3 | 2 | 44 |
| Linn BS 1968 | 1 | 2 | 5 | 4 | 1 | 0 | 13 |

**Reference:**

1.Kwong JCC, Khondker A, Lajkosz K, et al. APPRAISE-AI tool for quantitative evaluation of AI studies for clinical decision support. *JAMA Netw Open*. 2023;6(9):e2335377. https://doi.org/10.1001/ jamanetworkopen.2023.35377
